# Competing Substrate and Free-Surface Effects on Ion Transport in Ultrathin Polymer Electrolyte Films

**DOI:** 10.1021/acsmacrolett.6c00250

**Published:** 2026-08-21

**Authors:** Shannon Zhang, Ohad Bouni, Daniel Sharon, Michael A. Webb

**Affiliations:** † Department of Chemical and Biological Engineering, 6740 Princeton University, Princeton, New Jersey 08540, United States; ‡ Institute of Chemistry, 26742 The Hebrew University of Jerusalem, Givat Ram, Jerusalem, 9190401, Israel

## Abstract

Ceramic nanoparticles incorporated into solid polymer electrolytes have been reported to both enhance and suppress ionic conductivity, but the origins of these effects remain unclear due to difficulties in isolating interfacial phenomena in disordered bulk systems. Here, we characterize ion transport in ultrathin films of poly­(ethylene oxide)–lithium bis­(trifluoromethanesulfonyl)­imide on silica and employ analytical modeling to elucidate how interfaces might control thickness-dependent behavior. Ionic conductivity is suppressed for the thinnest films at all salt concentrations and temperatures, though modest enhancement appears in some films at low salt concentration. The extent of modulation, however, varies with concentration alone. A modified Vogel–Fulcher–Tammann model incorporating both substrate and free-interface effects on polymer dynamics best captures the observed behavior, outperforming models based on nonconducting layers, interfacial salt partitioning, or substrate-only interface effects. This indicates that both inorganic surfaces and free interfaces can substantially influence polymer mobility and conductivity, suggesting that strategies to optimize composite polymer electrolytes should address the coupled behavior of interfacial polymer dynamics.

## Graphical abstract

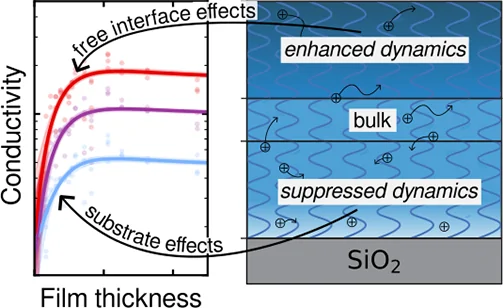

Composite polymer electrolytes (CPEs) contain fillers that modulate ionic conductivity, mechanical strength, and safety in solid-state batteries.^1,2^ Fillers can be active (ion-conducting), modulating conductivity directly by participating in and forming new ion transport pathways, or passive (non-ion-conducting), modulating conductivity by changing polymer structure, mobility, and ion solvation behavior.^3^ In homopolymers, ion transport occurs through ion hopping facilitated by polymer segmental motion^4–8^ and is limited by chelation and ion aggregation.^9–12^ The inclusion of passive ceramic fillers can either enhance or suppress conductivity.^13^ Enhancement above the polymer melting point is attributed to interfacial phenomena that locally facilitate ion mobility,^14–18^ while enhancement at room temperature is linked to suppression of polymer crystallinity.^15,19,20^ Conversely, other studies report conductivity suppression due to hindered segmental motion and elevated glass transition temperature (*T*_g_) at the polymer–filler interface.^14,21,22^ These competing effects remain poorly understood, largely because isolating interfacial phenomena in disordered bulk systems is difficult. Thin films offer a route to isolate interfacial contributions. Interdigitated electrodes (IDEs) provide enhanced sensitivity to surface effects in thin films^23,24^ and serve as proxies for interfacial behavior at controlled length scales.^25,26^ Dong et al. used this approach to quantify conductivity suppression in poly­(ethylene oxide)–lithium bis­(trifluoromethanesulfonyl)­imide (PEO–LiTFSI) films grafted onto silica, attributing the effect to an immobile interfacial zone.^24^ Additionally, although free-surface effects on polymer dynamics are well established,^27,28^ the inclusion of ions introduces additional complexity through polymer–ion coupling and ion–substrate interactions. Molecular dynamics simulations have begun to address these factors, revealing higher Li–TFSI coordination,^29^ reduced polymer mobility,^29,30^ and altered chain conformations^29,31^ near attractive surfaces, but bulk-like behavior near neutral substrates.^30^ Collectively, this literature demonstrates that interfacial effects vary substantially with substrate chemistry and interface type.

In this study, we examine interfacial effects on ion transport as induced by a passive substrate. In particular, we isolate, characterize, and present a consistent understanding of the effects of direct PEO–silica interactions on ionic conductivity in ultrathin films of PEO–LiTFSI. Silica is a commonly studied passive filler in PEO that is strongly attracted to PEO.^32–34^ Our films are fabricated to lie directly on the exposed oxide surface of the substrate, and the influence of interfacial effects is uncovered in increasing isolation by systematically decreasing film thickness. IDEs facilitate the measurement of ionic conductivity (Figure S1). Because the film-averaged conductivity from IDE measurements cannot directly resolve spatially inhomogeneous transport, we analyze a series of analytical models, each incorporating candidate physics in controlled ways to identify microscopic origins. We reveal a temperature-invariant suppression of ionic conductivity that reduces normalized conductivity by nearly 60% for thin films at low salt concentrations. The suppression is well-described by a modified Vogel–Fulcher–Tammann (VFT) model that accounts for both salt- and interface-induced modulation of polymer dynamics. These results highlight the nontrivial significance of optimizing polymer–substrate interactions based on their dynamic coupling for improved CPE performance.

We measured ionic conductivity in thin films with thicknesses *h* = 10–250 nm at salt concentrations *r* = [Li^+^]/[O] = 0.02, 0.05, and 0.10 and temperatures of 60, 80, and 100 °C, all well above the *T*_g_ of PEO (≈−60 °C).^35^ From DSC measurements, we determine that there is no residual film crystallinity in our samples (Figure S3). Decreasing film thickness is expected to amplify interfacial effects, while increasing *r* suppresses polymer dynamics.^11^

Figure 1 shows that conductivity generally decreases as films become thinner, indicating that interfacial interactions suppress ion transport; some films at low salt concentration exhibit modest enhancement above the bulk value. Variation in the measurements at nominally identical conditions is due to our measurements being drawn from independently fabricated samples rather than repeated measurements of the same device (see Section S1 for discussion of methods and sample-to-sample variability). To quantify the suppression, we define

1 $$\xi \left(r,T,h\right)=\left(\frac{\sigma^{\left(0\right)}\left(r,T\right)-\sigma \left(r,T,h\right)}{\sigma^{\left(0\right)}\left(r,T\right)}\right)\times 100\%$$

as the percent reduction relative to bulk-like conductivity σ^(0)^, which is the average conductivity of the thickest films studied (Section S4). For films with 10 nm ≤ *h* ≤ 20 nm, ξ reaches 59% at *r* = 0.02, 49% at *r* = 0.05, and 42% at *r* = 0.10, demonstrating that the relative influence of interfacial interactions diminishes with increasing salt concentration. Suppression is compared across a fixed thickness band (10 nm ≤ *h* ≤ 20 nm) rather than at each data set’s smallest available *h* so that the comparison is consistent across *r*.

**Figure 1:**
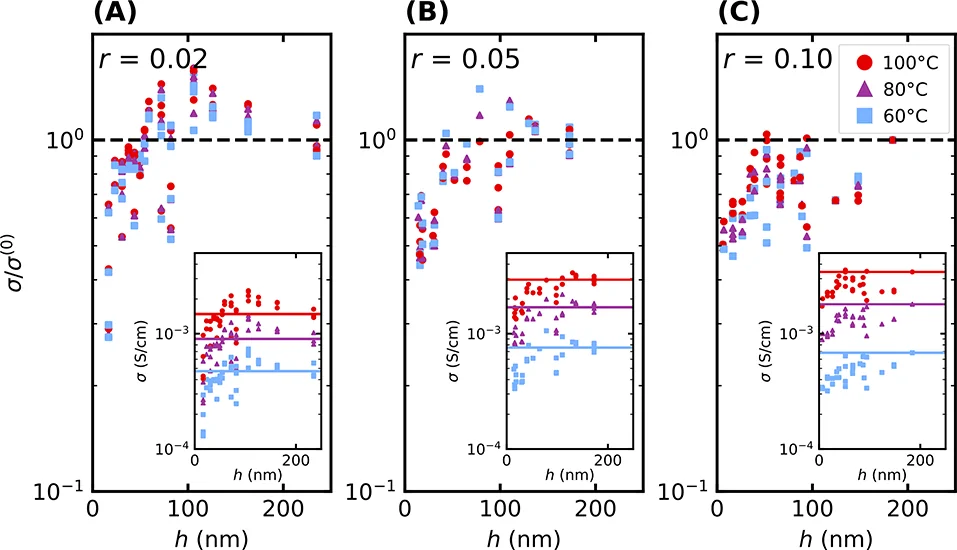
EIS-measured conductivity in silica-supported PEO–LiTFSI films. Conductivities normalized by σ^(0)^ are shown for films of varying thicknesses at concentrations of (A) *r* = 0.02, (B) *r* = 0.05, and (C) *r* = 0.10. Dashed lines are guides to the eye at σ = σ^(0)^. Insets show non-normalized conductivity measurements. Horizontal lines in the insets are σ^(0)^ and shaded regions around horizontal lines are standard deviations of σ^(0)^. The legend in (C) indicating data at different temperatures applies to all panels and insets.

By contrast, temperature does not notably influence the extent of interfacial suppression. Although absolute conductivity increases with temperature as expected for ion hopping facilitated by polymer segmental motion (Figure 1, insets), the normalized data collapse at each concentration (Figure 1, main axes), indicating that ξ is temperature-independent (see Section S2 for statistical testing). This suggests that, irrespective of any interface-induced changes to the underlying mechanisms, the interfacial transport process exhibits the same thermally activated functional form. Notably, the measured impedance spectra do not suggest a separate interfacial transport pathway as it exhibits a single semicircle with no distinct secondary relaxation; however, any such process would not be resolvable if its characteristic time scale were comparable to that of bulk-like transport.

To elucidate the physical origins of these trends, we adapt a phenomenological model for bulk polymer electrolyte conductivity to account for interfacial effects. We begin with the relationship proposed by Tsamopoulos et al.,^36^ with bulk conductivity expressed as

2 $$\sigma^{\left(0\right)}\left(r,T\right)=\lambda \left(T\right)r\frac{D_{p}\left(r,T\right)}{D_{p}\left(r=0,T\right)}$$

where λ is the molar conductivity at infinite dilution and *D*_p_ is the polymer center-of-mass diffusivity. The ratio *D*_p_(*r*, *T*)/*D*_p_(0, *T*) captures the coupling between ion mobility and polymer dynamics. Although the expression utilizes *D*_p_, it remains descriptive of coupling to segmental dynamics. This can be understood by noting that *D*_p_ relates inversely to the Rouse relaxation time τ_R_, which in turn sets the relaxation time of mode *p* as τ_p_ = τ_R_/*p* ^2^. The ratio *D*_p_(*r*, *T*)/*D*_p_(0, *T*) therefore recovers the same coupling whether expressed through center-of-mass diffusivity or through the relaxation time of any mode *p*, since the mode-number dependence cancels. This cancellation is consistent with prior findings that salt suppresses the dynamics of LiTFSI-doped PEO roughly uniformly across modes.^11^ The *D*_p_ is described by a modified VFT equation

3 $$D_{p}\left(r,T\right)=B\exp\left[\frac{-\left(E^{\left(0\right)}+E^{\left(1\right)}r\right)}{T-\left(T_{0}^{\left(0\right)}+a^{\left(1\right)}r\right)}\right]$$

where quantities superscripted with ‘(0)’ are neat-polymer properties and those with ‘(1)’ account for salt-concentration dependence. This model quantitatively reproduces bulk-like conductivity in our thickest films after parameter fitting (Section S4 and Figure S9).

To capture the thickness-dependent suppression observed in thinner films, we introduce modifications that account for interfacial phenomena. We consider five models grouped by physical picture. Two describe the interfacial region as a discrete layer, namely a nonconducting layer (NCL) and a salt-segregation layer (SSL). A third extends the latter by allowing the interfacial layer to follow a distinct ion-transport mechanism (SSLm). The remaining two modulate transport by altering polymer dynamics, either from substrate interactions alone (APD) or from both substrate and free-interface interactions (APD-FI). Fitting and then comparison to the data generated with the thin-film geometry provides a pathway to assess possible physics underlying observed trends. Across the latter four models, the interfacial parameters are physically motivated but represent phenomenological modifications of the local transport behavior rather than quantitatively established changes in any single microscopic quantity.

Parameter fitting is done with a two-step process where the Tree-structured Parzen Estimator (TPE) method is used to estimate baseline parameter values alongside an alternating optimization heuristic to account for any coupled parameters and regularization to penalize curvature in the parameter space with *r*. Then, the baseline values are used as central values for prior distributions in Bayesian parameter estimation (BPE)^37^ for refinement and estimation of parameter uncertainties (Section S6). Electrostatic screening is neglected because the Debye length (≈0.1 nm) is small enough that meaningful contributions are not expected (Section S3). We similarly neglect the effect of substrate surface charge.

*Nonconducting layer (NCL) model*: Our first adaptation treats a region of the film near the substrate as simply nonconducting (Figure 2A). In this NCL model, the measured conductivity is the bulk value scaled by the fraction of the film outside this region,

4 $$\sigma^{\mathrm{NCL}}\left(r,T,h\right)=\frac{\left(h-z_{1}\right)}{h}\sigma^{\left(0\right)}\left(r,T\right)$$

where *z*_1_ is the thickness of the nonconducting layer, assumed to be independent of film thickness. Although the physical picture envisions this region near the substrate, the equation captures only the effective nonconducting fraction and is agnostic to specific origins such as ion segregation or enhanced ion pairing.

**Figure 2:**
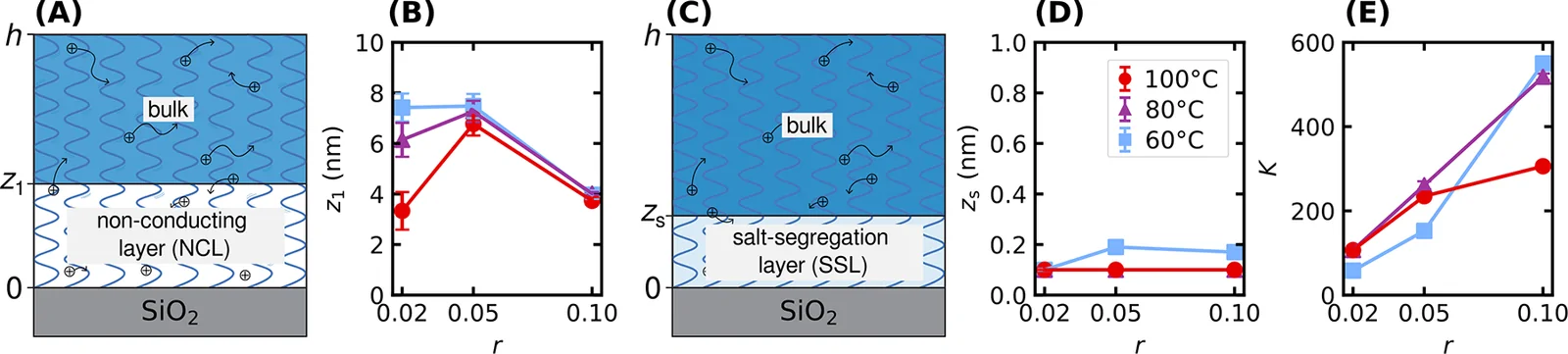
Nonconducting layer (NCL) and salt-segregation layer (SSL) model. Schematics illustrating envisioned manifestation of (A) NCL model with parameter *z*_1_ and (C) SSL model with parameter *z*_s_. The lightness of the blue shading indicates how conductive a region is, with white indicating no conductivity and dark blue indicating high conductivity. Dependence of (B) *z*_1_, (D) *z*_s_, and (E) *K* on salt concentration after fitting experimental data. Points are Bayesian parameter estimation posterior medians with error bars indicating 94% highest density intervals. The legend in (D) also applies to (B,E). Solid lines are guides for the eye.

Figure 2B shows that the values of *z*_1_ range from 3.3 to 7.3 nm, which are comparable to those reported in simulations for altered polymer conformations near attractive substrates^29,31^ and ion segregation near block copolymer interfaces.^38,39^ The values of *z*_1_ vary nonmonotonically with *r*, which is unexpected. We expect the decreasing trend from *r* = 0.05 to 0.10 because *z*_1_ is the incremental suppression arising from substrate proximity relative to the bulk, which diminishes as ion–polymer interactions increasingly suppress bulk dynamics at higher concentrations. We attribute the increase from *r* = 0.02 to 0.05, which is more prominent with increasing temperature, to experimental measurements that exceed σ^(0)^ that the NCL model cannot capture because it is implicitly bounded above by bulk conductivity. The frequency and magnitude of such measurements increase as concentration and temperature decrease (Figure S10), resulting in deteriorating model performance. The model is also limited by assuming uniform salt concentration throughout the film; preferential ion segregation to the interface would violate this assumption and complicate interpretation of *z*_1_.

*Salt-segregation (SSL) model*: To relax the assumption of uniform salt concentration, we consider preferential ion segregation in an interfacial region of thickness *z*_s_ (Figure 2C). In this model, a partitioning coefficient *K* relates the salt concentration in the interfacial region *r*_i_ to that in the bulk-like region *r*_b_ via

5 $$r_{i}=Kr_{b}$$

where *K* > 1 corresponds to interfacial enrichment. Conservation of salt across the film gives

6 $$r_{b}=\frac{hr_{0}}{h-z_{s}+Kz_{s}}$$

where *r*_0_ is the nominal salt concentration. The measured conductivity is then a thickness-weighted average of the two regions,

7 $$\sigma^{\mathrm{SSL}}\left(r_{0},T,h\right)=\frac{z_{s}}{h}\sigma_{i}\left(r_{i},T\right)+\frac{h-z_{s}}{h}\sigma^{\left(0\right)}\left(r_{b},T\right)$$

where σ_i_ is the conductivity of the interfacial region.

Figure 2D,E show the optimized values of *z*_s_ and *K*. The layer thickness *z*_s_ remains small and nearly constant across conditions, while *K* ≫ 1 indicates that a large excess of salt partitions into this thin interfacial layer, with *K* increasing with both salt concentration and temperature. Although increasing *K* with *r* is reasonable, the overall degree of partitioning is physically implausible. Because *K* and *z*_s_ jointly determine the film-averaged conductivity, these extreme values likely reflect the model compensating for its inability to capture the data rather than a genuine physical partitioning of salt.

*Modified salt-segregation (SSLm) model*: The poor description offered by the SSL model may stem from its assumption that interfacial conductivity follows the same physical picture as the bulk. The SSLm model relaxes this assumption and allows interfacial transport to be mechanistically distinct, though its specific nature is not determined here. Such a distinction may be plausible given prior simulations that, although conducted in different settings, revealed strong silanol-lithium interactions^40^ as well as interfacially altered polymer conformations,^41,42^ which can influence ion solvation and therefore transport near substrate surfaces. Alongside eqs 5 and 6, conductivity in the SSLm model is

8 $$\sigma^{\mathrm{SSLm}}\left(r_{0},T,h\right)=A\frac{z_{\mathrm{s,m}}}{h}\sigma^{\left(0\right)}\left(r_{i},T\right)+\frac{h-z_{\mathrm{s,m}}}{h}\sigma^{\left(0\right)}\left(r_{b},T\right)$$

where *A* modulates interfacial conductivity; other parameters are as previously defined.

The results in Figure 3 again favor small interfacial layers (*z*_s,m_ < 1 nm, Figure 3A) with strong, temperature-dependent salt aggregation (*K*_m_ ≫ 1, Figure 3B) and severely suppressed interfacial conductivity (*A* ≈ 0, Figure 3C). The interfacial conductivity relative to the bulk-like region is given by the dimensionless quantity *A*σ^(0)^(*r*_i_)/σ^(0)^(*r*_0_), for which values above unity indicate enhancement and values below unity indicate suppression. The near-total suppression recovered here follows from the highly salt-filled interfacial layer and shows that the SSLm model reproduces the behavior of the SSL model despite its additional degree of freedom, with comparable magnitudes of aggregation (Figure 2E). Moreover, *K*_m_ varies strongly with temperature, which is inconsistent with the temperature invariance of conductivity suppression observed experimentally (Figure 1). Thus, this formulation of the SSLm model is an unlikely physical representation of observed trends.

**Figure 3:**
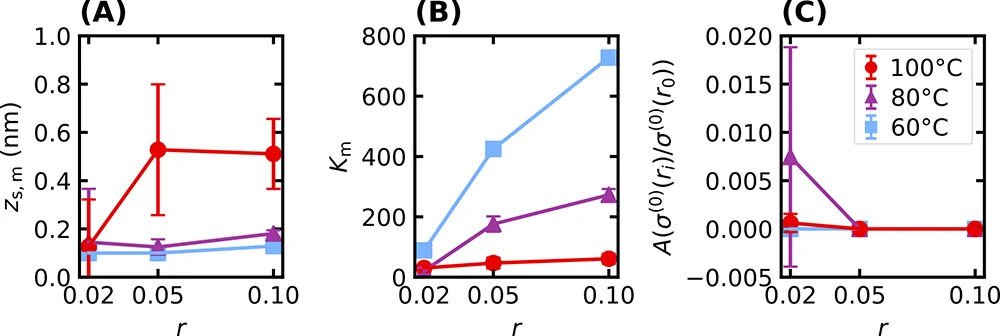
Modified salt-segregation layer (SSLm) model results. Dependence of (A) *z*_s,m_, (B) *K*_m_, and (C) *A* on salt concentration after fitting experimental data. Points are Bayesian parameter estimation posterior medians with error bars indicating 94% highest density intervals. The legend in (C) applies for all panels. Solid lines are guides for the eye.

*Altered polymer dynamics (APD) model*: In the APD model, we take a different approach from the previous models and probe how the substrate affects polymer dynamics, motivated by prior observations of modified glass-transition behavior near interfaces.^43–45^ This APD model introduces a position-dependent shift in the effective glass-transition temperature (Figure 4A):

9 $$D_{p}^{\mathrm{APD}}\left(r,T,z\right)=B\exp\left[\frac{-\left(E^{\left(0\right)}+E^{\left(1\right)}r\right)}{T-\left(T_{0}^{\left(0\right)}+a^{\left(1\right)}r+a^{\left(2\right)}\exp\left(-z/z^{\left(2\right)}\right)\right)}\right]$$

where *z* is the distance from the substrate. The new parameter *a* ^(2)^ characterizes the strength of substrate-induced dynamical suppression, while *z* ^(2)^ is the characteristic decay length of this perturbation into the film. Because polymer dynamics now vary continuously with position, the film-averaged conductivity is obtained by integration,

10 $$\sigma^{\mathrm{APD}}\left(r,T,h\right)=\frac{\lambda \left(T\right)r}{h}\int_{0}^{h}\frac{D_{p}^{\mathrm{APD}}\left(r,T,z\right)}{D_{p}\left(r=0,T\right)}dz$$

**Figure 4:**
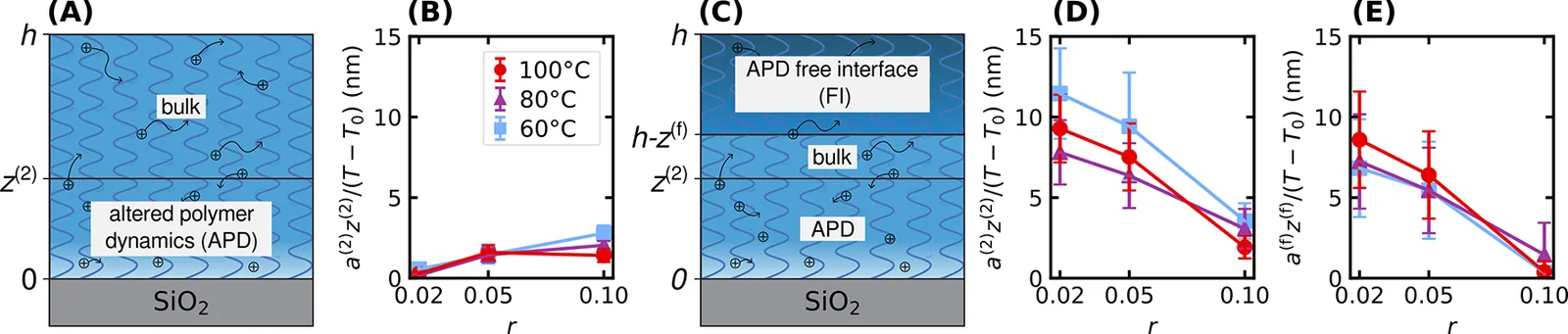
Comparison of models with altered polymer dynamics, both without (APD) and with (APD-FI) consideration of free-interface effects. (A, B) For the APD model, (A) a schematic representation of its parameters and (B) estimated effective penetration depths to characterize the influence of the substrate. (C–E) For the APD-FI model, (C) a schematic of its parameters and estimated effective penetration depths to characterize the influence of (D) the substrate and (E) the free interface. In (B), (C), and (E), the effective penetration depth is given by $\frac{a^{*}z^{*}}{T-T_{0}}$ , which is the integrated perturbation to the Vogel temperature, normalized by the thermal headroom. Points are Bayesian parameter estimation posterior medians with error bars indicating 94% highest density intervals. The lightness of the blue shading reflects conductivity, with white indicating low and dark blue indicating high conductivity. The legend in (B) also applies to (D) and (E). Solid lines are guides for the eye.

Figure 4B shows how the effective penetration depth *a* ^(2)^ *z* ^(2)^/(*T* – *T*_0_) varies with salt concentration. We examine the product *a* ^(2)^ *z* ^(2)^ rather than the individual parameters because a weaker local perturbation can be compensated by a longer decay length, whereas the product robustly approximates the integrated perturbation to the Vogel temperature across the film. Dividing by the thermal headroom *T* – *T*_0_ converts this perturbation into a length, interpreted as the depth over which the integrated substrate perturbation, if uniformly distributed, would equal the headroom for segmental motion. The actual perturbation extends deeper but with exponential decay. The penetration depth is independent of temperature and increases weakly with *r*. The temperature independence contrasts with the temperature sensitivity of the SSL model (Figure 2D). The weak increase with *r* is unexpected, since stronger ion–polymer suppression of bulk dynamics at higher concentrations should reduce the interface-to-bulk contrast and shorten the penetration depth. As with *z*_1_ in the NCL model, we attribute this trend to the model being implicitly bounded above by the bulk conductivity, which prevents it from capturing measurements that exceed σ^(0)^ at lower *r* and temperature (Figure S10).

*Altered polymer dynamics with free interface (APD-FI) model*: In the fifth model, we consider the effects of both the silica substrate and the free interface on polymer dynamics (Figure 4C). Free interfaces can modify glass-transition behavior as strongly as solid interfaces,^46–48^ and because they enhance polymer mobility, their inclusion relaxes the implicit upper bound of σ^(0)^ that constrained the previous models. The APD-FI model adds a term that enhances diffusivity near the free interface,

11 $$D_{p}^{\mathrm{APD}‐\mathrm{FI}}\left(r,T,z\right)=B\exp\left[\frac{-\left(E^{\left(0\right)}+E^{\left(1\right)}r\right)}{T-\left(T_{0}^{\left(0\right)}+a^{\left(1\right)}r+a^{\left(2\right)}\exp\left(\frac{-z}{z^{\left(2\right)}}\right)-a^{\left(f\right)}\exp\left(-\frac{\left(h-z\right)}{z^{\left(f\right)}}\right)\right)}\right]$$

where *a* ^(f)^ characterizes the strength of free-interface enhancement and *z* ^(f)^ is the corresponding decay length. Film-averaged conductivity is again obtained by integration.

Figure 4D,E show how the effective penetration depths from the substrate, *a* ^(2)^ *z* ^(2)^/(*T* – *T*_0_), and the free interface, *a* ^(f)^ *z* ^(f)^/(*T* – *T*_0_), vary with concentration and temperature. Because our aim is to discriminate among qualitatively distinct physical pictures of the interfacial region rather than to extract precise length scales, these values serve as convenient proxies. Both are temperature-independent and decrease with concentration, reflecting the diminished dynamical contrast between interfacial and bulk regions as ion–polymer interactions increasingly suppress mobility. Their magnitudes are comparable except at *r* = 0.10, where the free-interface contribution becomes negligible. Notably, including the free interface increases the inferred substrate perturbation relative to the APD model, indicating that the two interfaces compete. The optimized penetration depths are on the order of 10 nm, though the actual perturbations likely extend considerably farther given their exponential decay. Reported length scales for interfacial effects on polymer dynamics vary widely with the characterized property, ranging from a few to hundreds of nanometers,^22,46,49,50^ and direct comparison is complicated by the presence of salt in our systems and by differences in how the length scales are defined.

*Model comparison*: Figure 5A compares model performance using the expected log pointwise predictive density (ELPD),^51^ which measures how well the posterior distribution estimated from the BPE parameters predicts a given set of observations, with higher values indicating better performance. We evaluate ELPD across all log-transformed conductivity measurements (Section S7). The SSLm model performs worst, consistent with the physical inconsistencies identified above, and the NCL and SSL models offer only minor improvement. The APD and APD-FI models supply substantially better representations of the data, with APD-FI achieving the best performance overall but statistically similar to APD in terms of aggregate ELPD. This indicates that a continuous modification of polymer dynamics provides a more physically consistent description than effectively nonconducting or simple salt-segregating layers. Nevertheless, neither the conductivity measurements alone nor their (in)­consistency with these models can determine whether altered polymer dynamics, ion partitioning, ion association, or some combination thereof produce the observed thickness dependence.

**Figure 5:**
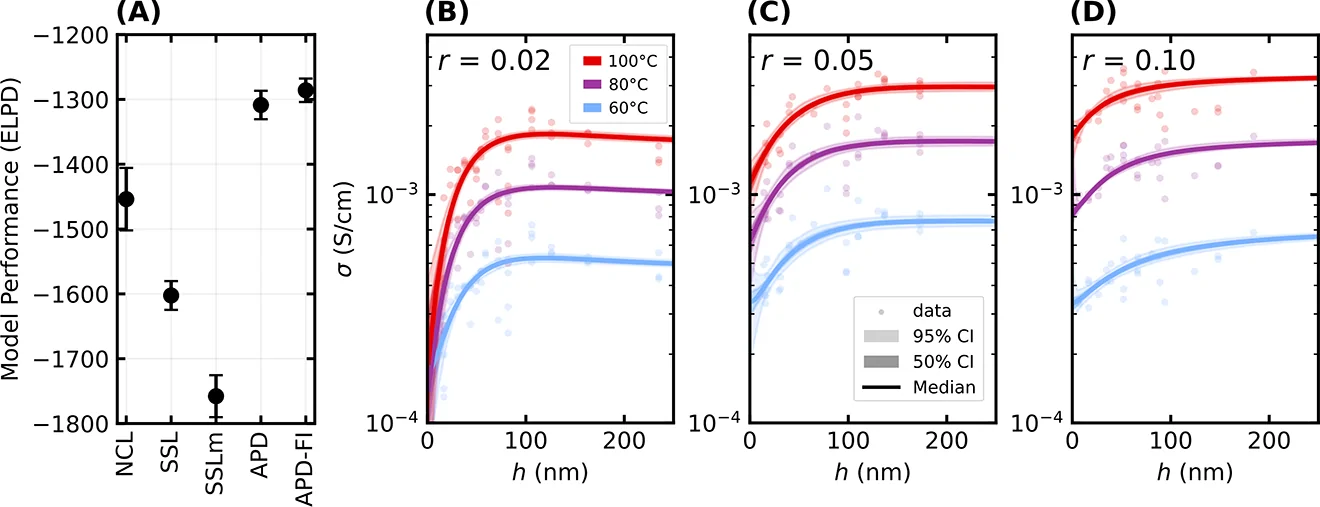
Model comparison. (A) Comparison across all models using expected log pointwise predictive density (ELPD). Error bars represent the standard error calculated over all experimental measurements of conductivity. (B–D) Comparison of APD-FI model results to experimental data. Conductivities are shown for films of varying thicknesses at concentrations of (B) *r* = 0.02, (C) *r* = 0.05, and (D) *r* = 0.10. Transparent points are experimental data points, which are identical to the points in the inset of Figure 1. Solid lines are model results using median values of the optimized parameter posteriors and shaded regions are 50% (light) and 95% (dark) credible intervals. The legend in (B) indicating data at different temperatures and in (C) applies to all panels (B–D).

The marginal improvement of APD-FI over APD arises from the two competing interfacial terms. The free-interface term enhances local mobility and thereby relaxes the upper bound of σ^(0)^ that constrains the other models, allowing it to capture the modest conductivity enhancement observed in some thin films. The competition also improves the description of the steep decrease in conductivity at small *h* relative to the substrate term alone. Stratifying ELPD by film thickness reveals that this advantage is localized to the thinnest films (Figure S28). Given the substantial sample-to-sample variability in some thickness regimes (Figure S2), whether this added flexibility is necessary to capture a genuine interfacial enhancement warrants further investigation. Nevertheless, the inclusion of both effects may be reasonable given prior reports that free-interface and substrate contributions are comparable for quantities like *T*_g_.^46–48^ Ultimately, both APD and APD-FI frameworks provide consistent phenomenological descriptions of the experimental conductivity trends.

Visual comparison of model predictions to experimental data illustrates the quality of the APD-FI fits (Figure 5B–D), with the remaining models compared in Figures S23–S27. While ELPD provides one means of assessment, the relative rankings are effectively corroborated by considering an alternative metric based on the coefficient of determination (Figure S29, Section S7). This measure places the APD-FI model well ahead of APD, and both outperform the remaining three, rejected models, which is consistent with the interpretation of ELPD. It is worth noting that all models neglect more nuanced interfacial perturbations to ion solvation, which could influence conductivity and salt solubility and may differ between the substrate and free interface owing to the distinct chain conformations established at each.^41,42^ We expect these effects to be localized to short length scales relative to even our thinnest films and thus to weakly influence the film-averaged behavior probed here, though this likely depends on system and warrants future study.

In summary, we showed how ionic conductivity in ultrathin polymer electrolyte films can be phenomenologically described by coupling transport to interfacially altered polymer dynamics, considering both substrate and free-interface effects. Between models that account for substrate-only versus dual-interface effects, the latter provides extracted length scales that are closer to those reported for interfacial effects on polymer *T*_g_. Neither model explicitly nor uniquely identifies underlying microscopic origins for these perturbations, however, and sample-to-sample variability in our measurements obfuscates clean discrimination. This motivates future studies that can more definitively establish whether conductivity is genuinely enhanced at lower concentrations in the thinnest films or whether other mechanistic factors are at play. The existence of interfacial coupling would have direct implications for composite polymer electrolytes, since filler loadings could create confinement lengths comparable to the perturbation depths identified here. Similar considerations would apply to block copolymer electrolytes, where domain spacings may be 10–50 nm.

## Supplementary Material



## Data Availability

The data underlying this study are available in the published article and its Supporting Information.

## Notes

Experimental methods: (Materials; Interdigitated electrode device fabrication; Experimental ionic conductivity measurements using EIS; Differential Scanning Calorimetry); Temperature independence of normalized experimental samples; Calculation of Debye length; Calculation of bulk conductivity; Experimental measurements above bulk conductivity; Optimization of model parameters; Model comparison (PDF)
