# Supplementary material for: Competing Substrate and Free-Surface Effects on Ion Transport in Ultrathin Polymer Electrolyte Films
Source: ACS Macro Lett. 2026 Aug 21;15(9):1208–14. doi: 10.1021/acsmacrolett.6c00250 (PMC13585131; doi:10.1021/acsmacrolett.6c00250)
Supplement: Supplementary file 1 [file mz6c00250_si_001.pdf]

Supporting Information
Competing Substrate and Free-Surface Effects on Ion Transport in Ultrathin Polymer Electrolyte Films

Shannon Zhang,[†,¶] Ohad Bouni,[‡,¶] Daniel Sharon,[*,‡] and Michael A. Webb[*,†]

†*Department of Chemical and Biological Engineering, Princeton University, Princeton, NJ, 08544 USA*

‡*Institute of Chemistry, The Hebrew University of Jerusalem, Givat Ram, Jerusalem, 9190401 Israel*

¶*These authors contributed equally to this work.*

E-mail: daniel.sharon@mail.huji.ac.il; mawebb@princeton.edu

Contents

S1 Experimental methods **SI-2**
S1.1 Materials . . . SI-2
S1.2 Interdigitated electrode device fabrication . . . SI-2
S1.3 Experimental ionic conductivity measurements using EIS . . . SI-2
S1.4 Differential Scanning Calorimetry . . . SI-3

S2 Temperature independence of normalized experimental samples **SI-6**

S3 Calculation of Debye length **SI-8**

S4 Calculation of bulk conductivity **SI-10**

S5 Experimental measurements above bulk conductivity **SI-14**

S6 Optimization of model parameters **SI-15**
S6.1 Initial parameter estimation using TPE . . . SI-15
S6.2 Parameter refinement and uncertainty estimation using BPE . . . SI-23

S7 Model Comparison **SI-34**

References **SI-36**

S1 Experimental methods

S1.1 Materials

Polyethylene oxide (PEO, Mw 20,000 Da) was dissolved in anhydrous acetonitrile purchased from Sigma-Aldrich. Lithium bis(trifluoromethanesulfonyl)imide (LiTFSI) salt was purchased from Gelion PLC and added to the PEO solution at the required concentration. Solution preparation was carried out under dry conditions to minimize water contamination.

S1.2 Interdigitated electrode device fabrication

Interdigitated electrode devices (IDEs) were fabricated on thermally treated 4-inch silicon wafers coated with a 1 micrometer silicon oxide layer. The electrode pattern was defined by photolithography using an MA6 mask aligner. The electrodes consisted of a 10 nm titanium adhesion layer and a 50 nm gold layer. The IDE included an electrode spacing d=8 μm, an electrode length ℓ=1 mm and an electrode width w=2 μm.[1] Figure S1 shows an illustration of the IDE design.

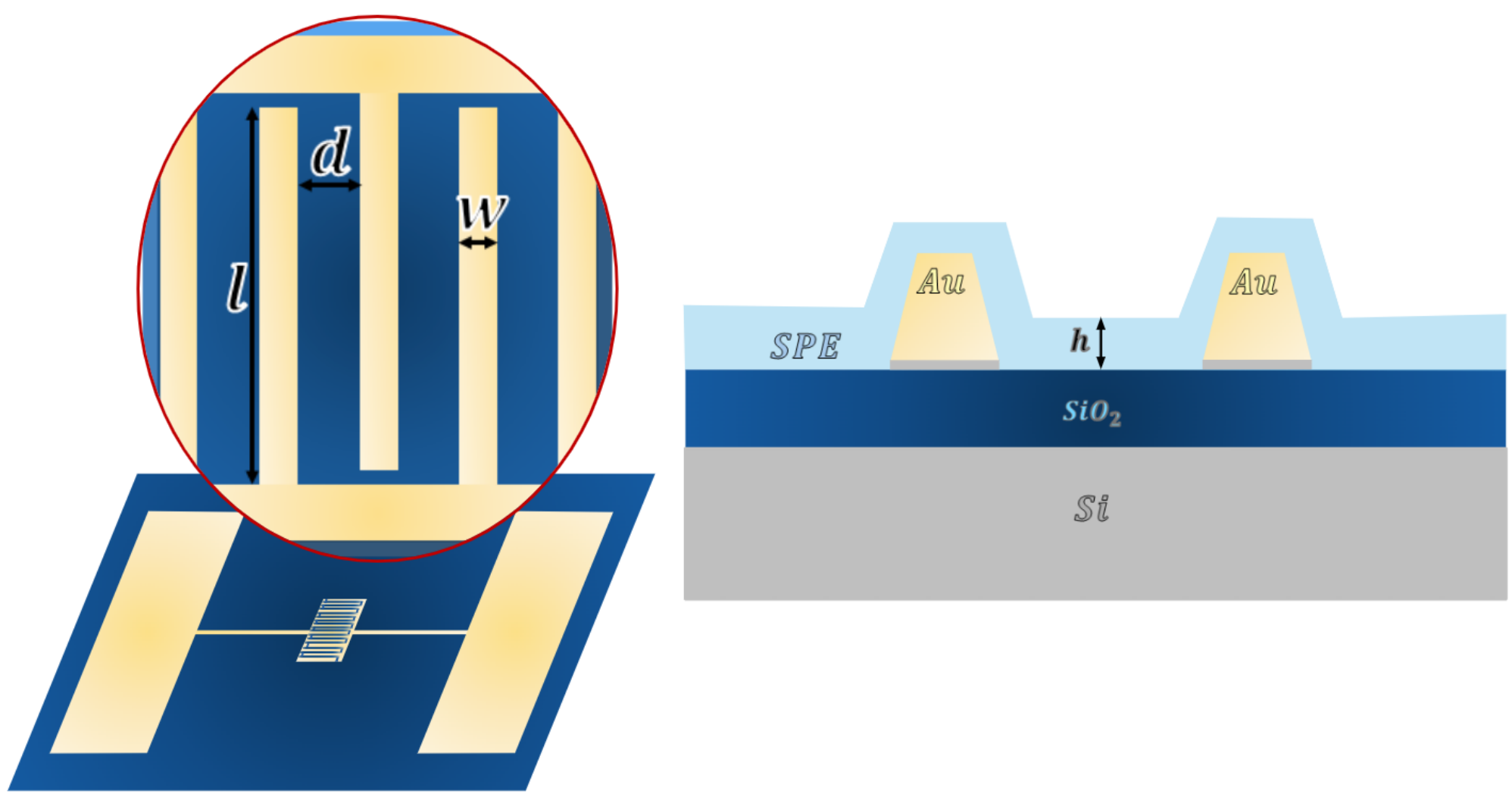

Figure S1: Illustrations of the IDE design where ℓ is electrode length, d is the distance between electrodes, w is electrode width, and h is PEO film thickness.

S1.3 Experimental ionic conductivity measurements using EIS

The ionic conductivity of the PEO-LiTFSI thin films was determined from electrochemical impedance spectroscopy (EIS) measurements performed on the coated IDEs. EIS measurements were taken in an IDE using the Gamry Reference 620 potentiostat inside an argon-filled glovebox and analyzed using an equivalent circuit model to extract the ionic resistance of each film. The impedance spectra were fitted using an equivalent circuit model. The ionic film resistance R was determined from the high-frequency intercept on the real axis and converted to conductivity based on the geometry of the electrode array. Using this R, the

ionic conductivity σ of the SPE was calculated using

$$\sigma = \frac{1}{R} \cdot \frac{d}{\ell(N-1)h} \tag{S1}$$

where d is the electrode gap (10 µm), ℓ is the electrode length (1 mm), N is the number of electrodes (160), and h is the SPE film thickness.

Scatter in our conductivity data (Figures 1 and S6) is due to our measurements being taken from independently fabricated samples rather than repeated measurements of the same device. Each data point represents a minimum of three distinct IDE devices fabricated across different batches. From these measurements, experimental variance stems from our determination of film thickness. The reflective gold patterns on the IDE devices interfere with standard ellipsometry models, so film thickness cannot be measured directly on the active devices. Instead, it is characterized using unpatterned silicon reference blanks (with three measurements taken across two distinct chips) that are spin-coated simultaneously alongside the IDE devices using the same solution and parameters. Despite identical settings, minute manual variations during spin-coating—such as a millisecond delay in solution deposition—can induce subtle chip-to-chip variations in the resulting film thickness. Since our ionic conductivity calculation is inversely proportional to and normalized by the film thickness, these minor physical variations directly propagate into the calculated conductivity values, manifesting as the observed data spread. Rather than omitting or smoothing data points that exhibited larger deviations, we elected to plot all individual experimental measurements to maintain complete scientific transparency regarding the true variance of the thin-film system. Quantification of the spread of our data is given in Figure S2, which shows the number of independently fabricated samples and coefficient of variation of nominally identical samples (same r, T, and h). The coefficient of variation is defined as the standard deviation over the mean of nominally identical samples. Larger values indicate larger sample-to-sample variation. Excluding measurements for which only a single nominally identical sample was tested, the coefficient of variation values are highest at $r = 0.02$ and low h, while the majority of our data remain ≤ 0.2. This reflects the increased difficulty of conductivity measurements at low h and the generally low variability elsewhere in the dataset.

S1.4 Differential Scanning Calorimetry

Differential scanning calorimetry (DSC) measurements were performed using a TA Instruments DSC250. PEO-LiTFSI and PEO samples were dried at 50°C under vacuum overnight to remove residual acetonitrile. Samples with masses of 10-15 mg were sealed in hermetic aluminum pans. The samples were first heated to 110°C to obtain uniform contact between the solid polymer electrolyte and the aluminum pan. The samples were then scanned from −90°C to 110°C at a heating rate of 15°C min^{-1}. Results are shown in Figure S3. Pristine PEO (20 kDa) displays a sharp melting onset at 62.16°C, which shifts to lower temperatures and flattens out with increasing salt content. This suppression of PEO crystallinity is attributed to the local coordination of cations along the polymer backbone and the steric hindrance imposed by the bulky counter-anions.[2]

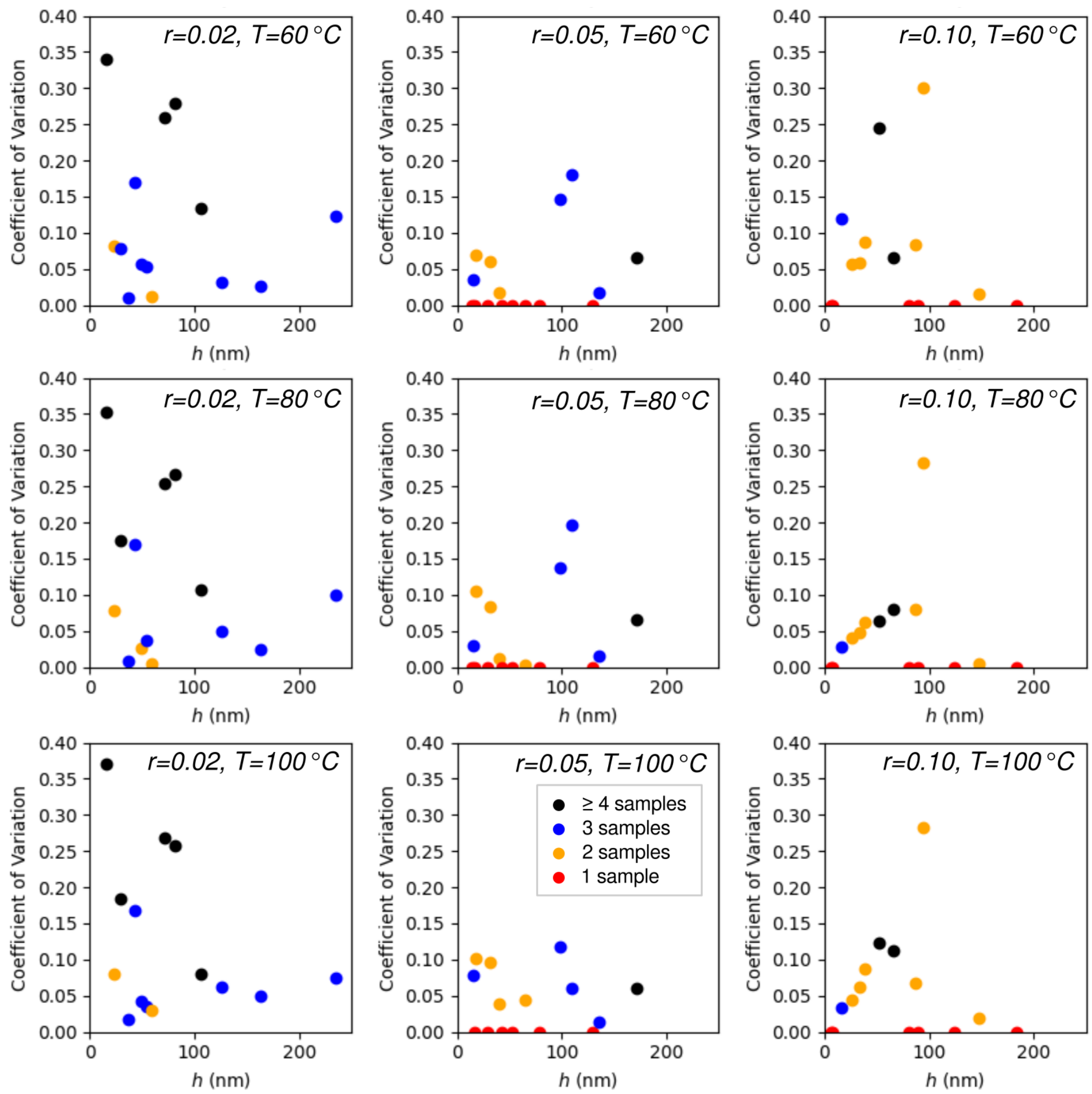

Figure S2: Variation in experimental data given as the coefficient of variation of nominally identical (same r, T, and h) samples. The legend in the bottom center panel indicating the number of identical samples at each point applies to all panels.

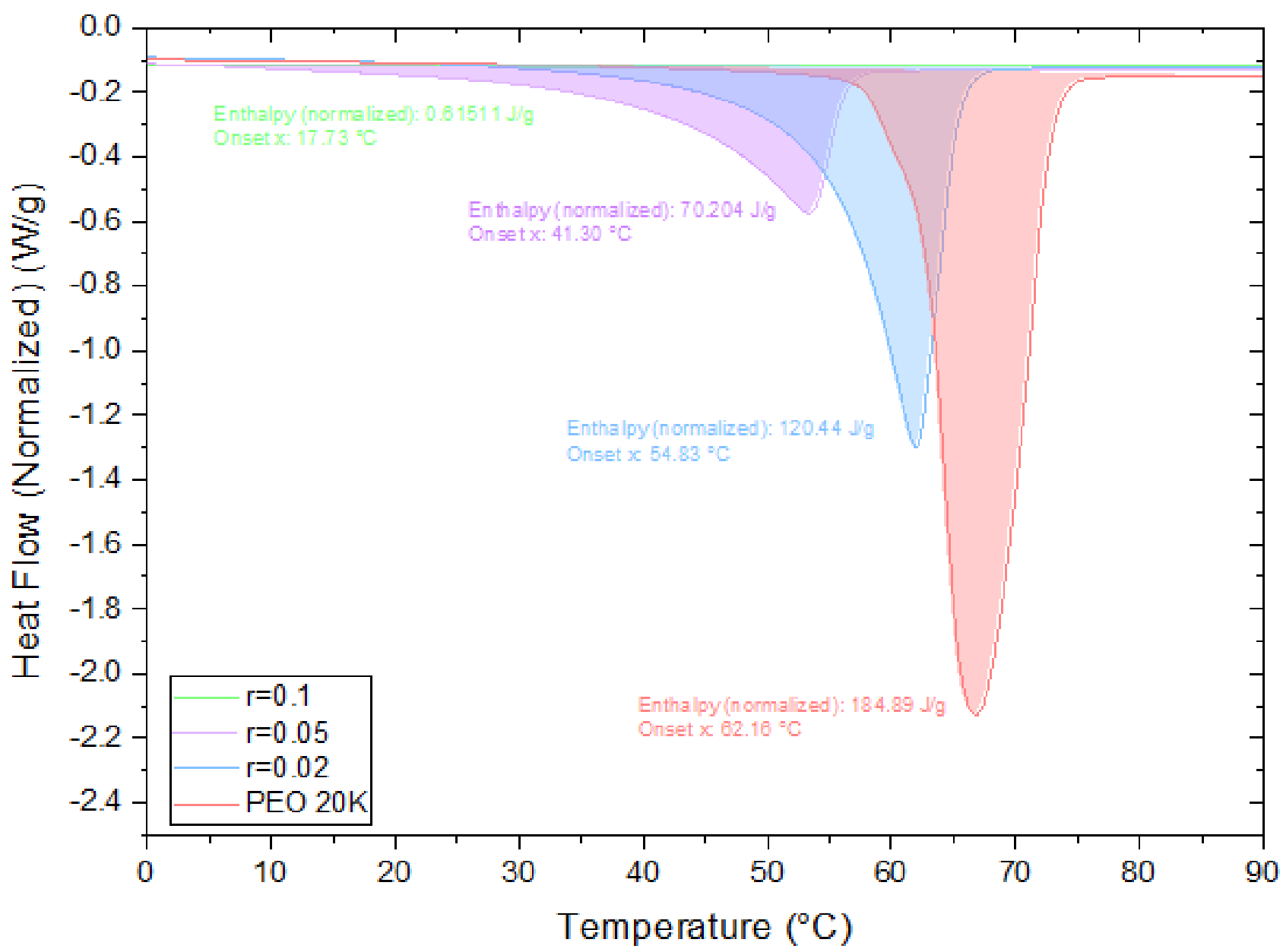

Figure S3: DSC heating curves (−90°C to 110°C at 15°C min^{-1}) showing the effects of LiTFSI concentration on the crystalline melting behavior of PEO.

S2 Temperature independence of normalized experimental samples

To evaluate whether our normalized experimental conductivity measurements exhibit statistically significant temperature dependence, we partition the data at each r into 10 nm bins starting from 0 nm. A one-way analysis of variance (ANOVA) is then applied within each bin. The test statistic for ANOVA is

$$F = \frac{\text{Variance between groups (VB)}}{\text{Variance within groups (VW)}} \tag{S2}$$

where groups within each bin are distinguished by temperature. VB and VW are defined as

$$\text{VB} = \frac{\sum_{i=1}^{k} n_i(\bar{x}_i - \bar{x})^2}{k-1} \tag{S3}$$

$$\text{VW} = \frac{\sum_{i=1}^{k}\sum_{j-1}^{n_i}(x_{ij} - \bar{x}_i)^2}{N-k} \tag{S4}$$

where k is the number of groups, n_i is the number of samples in group i, N is the total number of samples in the bin, $\bar{x}_i$ is the mean of all samples in group i, $\bar{x}$ is the mean of all samples in the bin, and x_{ij} is the jth sample in the ith bin.

To determine whether the observed variance between groups is significant relative to the inherent variability within the data (i.e., whether there is statistically significant temperature dependence), the calculated F-statistic is compared against the theoretical F-distribution with $k-1$ numerator degrees of freedom and $N-k$ denominator degrees of freedom. The resulting p-value is obtained from the area in the tail of the F-distribution beyond the observed F-statistic. The data is determined to be independent of temperature if the resulting p-value is greater than 0.05.

We show both the F-statistic and p-values of each bin at each r in Figure S4 The results suggest that the normalized data is statistically independent of temperature ($p > 0.05$) for all cases except at $r = 0.10$, bin $h =$(30, 40). Upon inspection of the raw data in Figure 1, the conductivity of samples in these bins is uniformly greater at 100°C than at 60°C. However, since $p > 0.05$ for all other bins, we conclude that the observed conductivity trends are mostly independent of temperature.

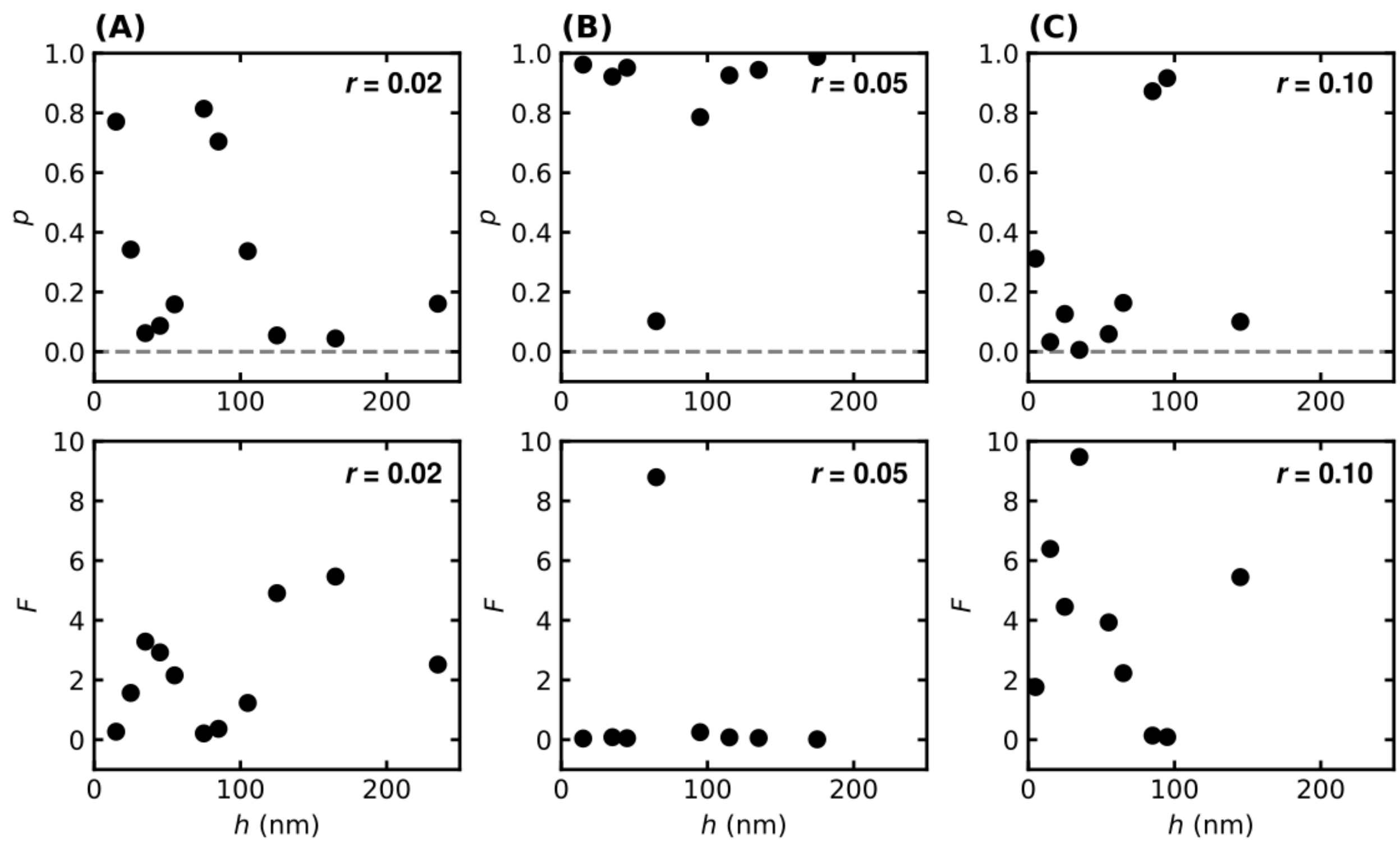

Figure S4: One-way ANOVA evaluation of experimental data. Results are shown as p-values in the top row and F-statistics in the bottom row for all experimental data at (A) $r = 0.02$, (B) $r = 0.05$, and (C) $r = 0.10$. The dashed line at $p = 0.05$ is a guide for the eye.

S3 Calculation of Debye length

The Debye length (λ_{D}) is estimates the size of the double layer and effect of electrostatic screening in our films. It is calculated using

$$\lambda_{\mathrm{D}} = \left(\frac{\epsilon k_{\mathrm{B}} T}{\sum_{j=1}^{N} n_j^0 q_j^2} \right)^{1/2} \tag{S5}$$

where ϵ is the permittivity of PEO, k_{B} is the Boltzmann constant, T is temperature, n_j is the number density of ion species j, and q_j is the charge of ion species j. For $\epsilon = \epsilon_r \epsilon_0$, where ϵ_r is the relative permittivity of PEO and ϵ_0 is the permittivity of a vacuum, we use an approximate ϵ_r value of 5,[3] but this can change with temperature. To calculate n, we first convert r to moles of salt per kg electrolyte (c) using a relationship fitted to results from existing experimental data,[4] which is shown in Figure S5. Assuming no volume change upon mixing of PEO and LiTFSI, we then convert c to moles of salt per liter electrolyte (M) using experimental mass densities of 1.12 g/cm^{-3} for PEO and 2.023 g/cm^{-3} for LiTFSI, respectively.[5] Calculation of n from the molarity is then trivial. An example calculation of n for $r = 0.10$ is as follows:

First, we calculate c from r

$$r = 0.10 \tag{S6}$$

$$c = 22.61/(10 + 6.47) = \frac{1.37 \text{ mol electrolyte}}{1 \text{ kg electrolyte}} \tag{S7}$$

Next, we calculate the mass (m) and volume (V) of LiTFSI and PEO assuming a total mass of 1 kg

$$1.37 \text{ mol LiTFSI} \times \frac{287.09 \text{ g LiTFSI}}{1 \text{ mol LiTFSI}} = 0.394 \text{ kg LiTFSI} \tag{S8}$$

$$0.394 \text{ kg LiTFSI} \times \frac{0.001 \text{ L LiTFSI}}{0.002023 \text{ kg LiTFSI}} = 0.195 \text{ L LiTFSI} \tag{S9}$$

$$1 - 0.394 \text{ kg LiTFSI} = 0.606 \text{ kg PEO} \tag{S10}$$

$$0.606 \text{ kg PEO} \times \frac{0.001 \text{ L PEO}}{0.00112 \text{ kg PEO}} = 0.541 \text{ L PEO} \tag{S11}$$

Then, we calculate the density ρ of the system

$$\rho = \frac{m_{\mathrm{LiTFSI}} + m_{\mathrm{PEO}}}{V_{\mathrm{LiTFSI}} + V_{\mathrm{PEO}}} = 1.36 \frac{\text{kg electrolyte}}{\text{L electrolyte}} \tag{S12}$$

Finally, we calculate the molarity of the system using ρ and c.

$$M = c \times \rho \tag{S13}$$

$$= 1.37 \frac{\text{mol electrolyte}}{\text{kg electrolyte}} \times 1.36 \frac{\text{kg electrolyte}}{\text{L electrolyte}} \tag{S14}$$

$$= 1.865 \frac{\text{mol electrolyte}}{\text{L electrolyte}} \tag{S15}$$

To calculate n from M, multiply M by Avogadro's number.

Calculated values of molarity and λ_D for r = 0.02, 0.05, and 0.10 at 100°C are provided in Table S1.

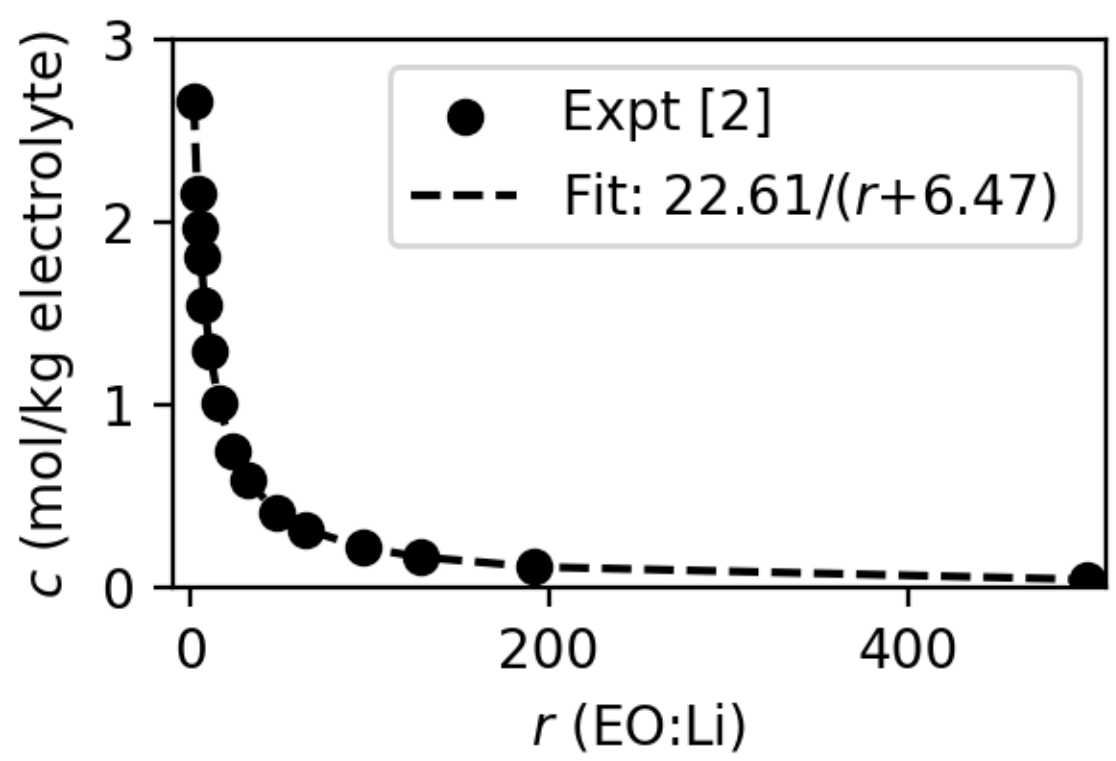

Figure S5: Relationship between r and moles of salt per kg electrolyte (c) derived from experimental results in Ref. 4. The dashed black line is a fit to the experimental conversion between r and c.

Table S1: Debye length (λ_D) and salt concentration of PEO-LiTFSI films in different units. Results are shown at T = 100°C and using a relative permittivity of PEO of $\epsilon_r = 5$.

r	c (mol/kg)	M (mol/L)	λ_D (nm)
0.02	0.40	0.47	0.12
0.05	0.85	1.30	0.074
0.10	1.37	1.87	0.062

S4 Calculation of bulk conductivity

To quantify the extent of conductivity suppression in our thin films, we first calculate an apparent bulk conductivity ($\sigma^{(0)}$) by averaging over the conductivity values measured for the thickest films in our experimental data from in Figure 1. To determine whether the conductivity values at these thicknesses are representative of bulk conductivity, we conducted a series of conductivity measurements separate from our original data in Figure 1 for films at $r = 0.02$ and 100°C, 80°C, and 60°C with h between 30 and 345 nm. This data is shown in Figure S6 as opaque data points overlaid on top of our original dataset at $r = 0.02$ from Figure 1 as transparent data points. There is an evident systematic shift in the original and new datasets. We attribute this to a known artifact of conducting an independent experimental campaign, since the new data were collected using a different spin-coater, probe station, IDE substrate batch, and polymer-salt solution. Such hardware and sample variations are well known to shift the absolute impedance between campaigns while preserving relative trends. Because of this, we compare interfacially-affected and bulk-like measurements only within each dataset and never across the two campaigns in aggregate. The new measurements are used solely to establish that a conductivity plateau is reached in sufficiently thick films, while all model fitting for a given condition uses self-consistent within-dataset bulk references. As a result, the new dataset is only used for this bulk h determination. All other analysis in the paper is done for the original dataset shown fully in Figure 1.

From Figure S6, it is visually apparent that conductivity plateaus in the thickest films measured. Quantitatively, the average conductivity deviation from the thickest films ($h =$ 345 nm) across all temperatures is 2.3%, 4.3%, 5.8%, and 19% for films with thicknesses of $h = 295$, 270, 198, and 167 nm, respectively. Exact values of average conductivities at each temperature and h at these conditions are provided in Table S2. Thus, we assume that our films take on bulk-like (plateaued) conductivity for all $h \geq 198$ nm, where the deviation in conductivity from the thickest film is less than 10%. In our original dataset, conductivity measurements are taken as bulk-like for films with $h = 235$ nm at $r = 0.02$, $h = 172$ nm at $r = 0.05$, and $h = 184$ nm and $r = 0.10$. Although the h values approach the interfacially modulated regime for $r = 0.05$ and $r = 0.10$, we assume that their deviation from bulk behavior remains small and note that this assumption may lead to a moderate overestimation of h.

Table S2: Change in average experimental conductivity with film thickness. Conductivity is given with units of S/cm.

h (nm)	$\langle\sigma(100°C)\rangle \times 10^3$	$\langle\sigma(80°C)\rangle \times 10^3$	$\langle\sigma(60°C)\rangle \times 10^3$
345	0.935	0.592	0.333
295	0.976	0.600	0.330
270	0.928	0.575	0.302
198	1.020	0.636	0.337
167	1.180	0.720	0.365

For all phenomenological models, an alternate measure of bulk conductivity is estimated by fitting the experimentally measured and averaged values of $\sigma^{(0)}$ to the original VFT model for conductivity in bulk polymers.[6] This method uses Equations 2 and 3 where B is a free

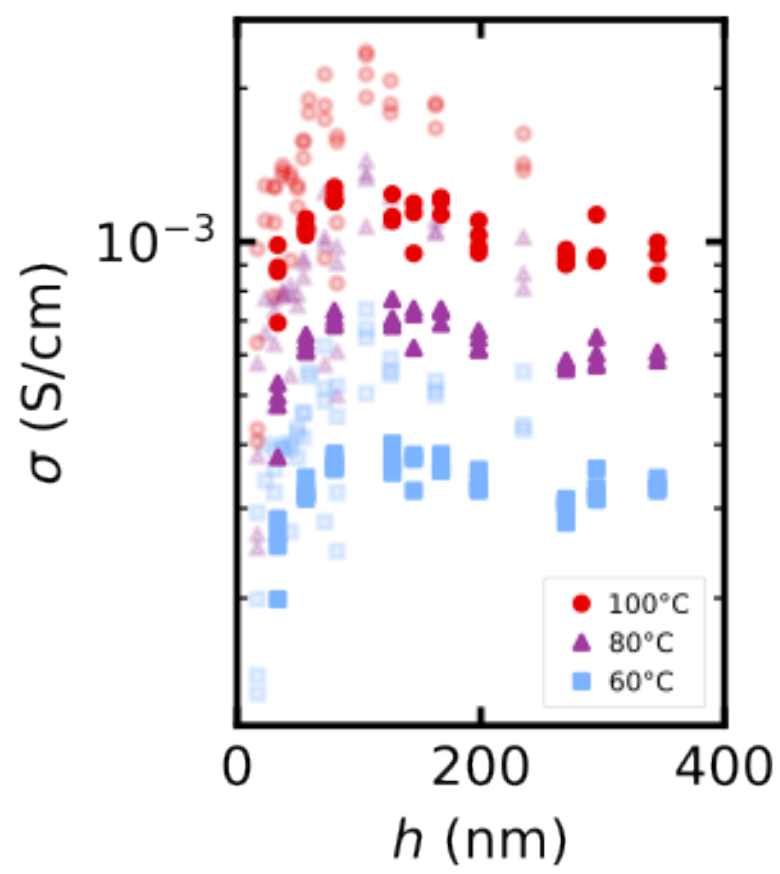

Figure S6: Experimental data at $r = 0.02$. The analyzed data also found in Figure 1 is shown as transparent points and alternate data used only for bulk conductivity estimation is shown as opaque points.

parameter, λ is the ionic conductivity in bulk PEO at infinite dilution, c_s is salt concentration, D_{P} is the polymer center of mass diffusivity, $E^{(0)}$ is the activation energy of pure PEO, T_0 is the Vogel temperature $T_{\mathrm{g}} - 50$°C where T_{g} is the glass transition temperature of pure PEO, and $E^{(1)}$ and $a^{(1)}$ are fitted parameters that quantify the dependence of the activation energy and T_{g} of PEO on salt concentration. To fit D_{P}, a value of $T_0 = 155$ K, determined by Niedzwiedz et al. for short PEO chains, is used.[7] Experimental values of T_{g} versus r shown in Figure S7 are used to fit $a^{(1)} = 127$.[8] Experimental values of D_{P} at a range of r values are shown in Figure S8, which are used to fit $E^{(0)} = 1451$, $E^{(1)} = 1250$, and $B = 0.000213$.[9] Finally, λ is fit to our bootstrapped values of $\sigma^{(0)}$ versus r at each temperature for $\lambda(60°\mathrm{C}) = 0.029$ S/cm, $\lambda(80°)\mathrm{C} = 0.058$ S/cm, and $\lambda(100°\mathrm{C}) = 0.093$ S/cm. With the provided fitting process and parameters, this model is able to quantitatively capture experimental trends for ionic conductivity in bulk PEO-LiTFSI systems, as shown in Figure S9.

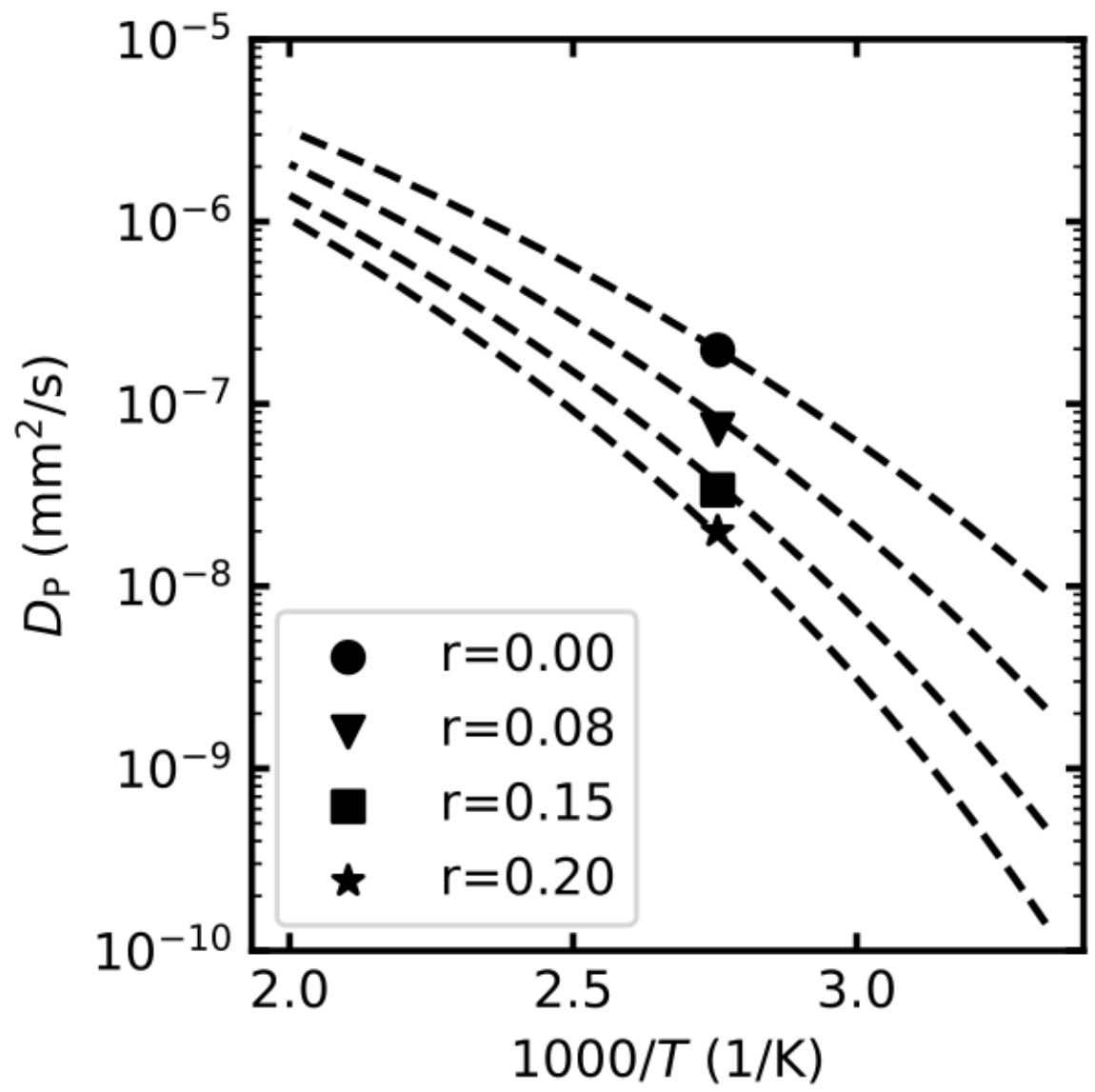

Figure S7: Experimental results[9] of PEO center of mass diffusivity D_{P} versus temperature in a range of salt concentrations and their fits. Dashed black lines are fits to the experimental data determined by varying $E^{(0)}$, $E^{(1)}$, and B in Equation 3.

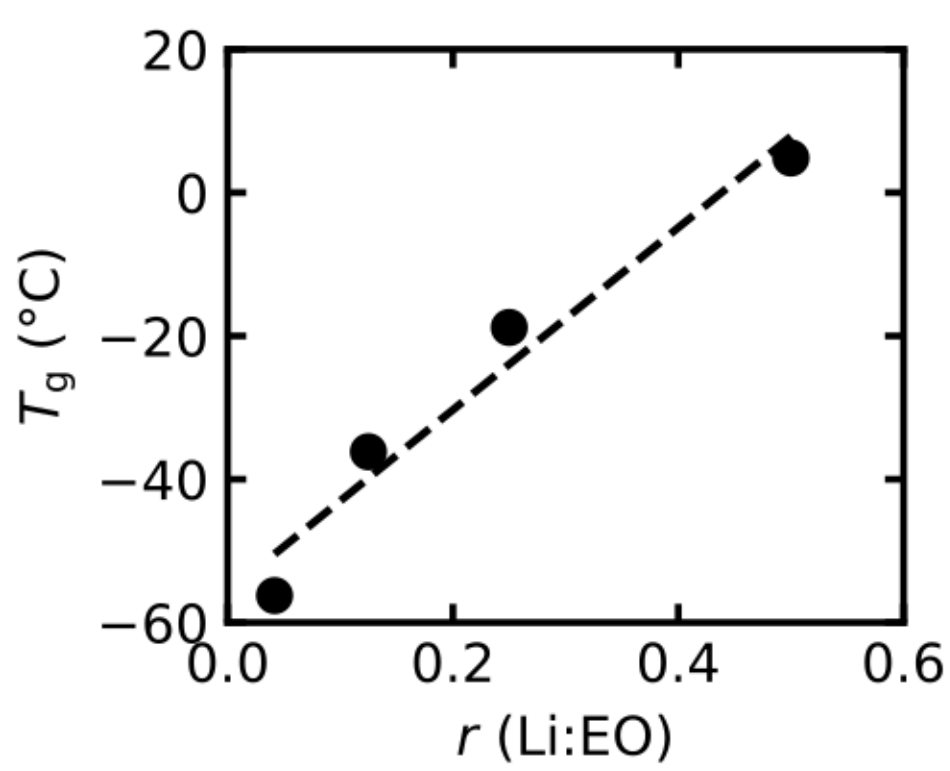

Figure S8: Experimental results[8] of PEO T_{g} versus r and their fit. The dashed black line is a linear fit to the experimental data of $T_{\mathrm{g}} = 127.13r - 55.73$.

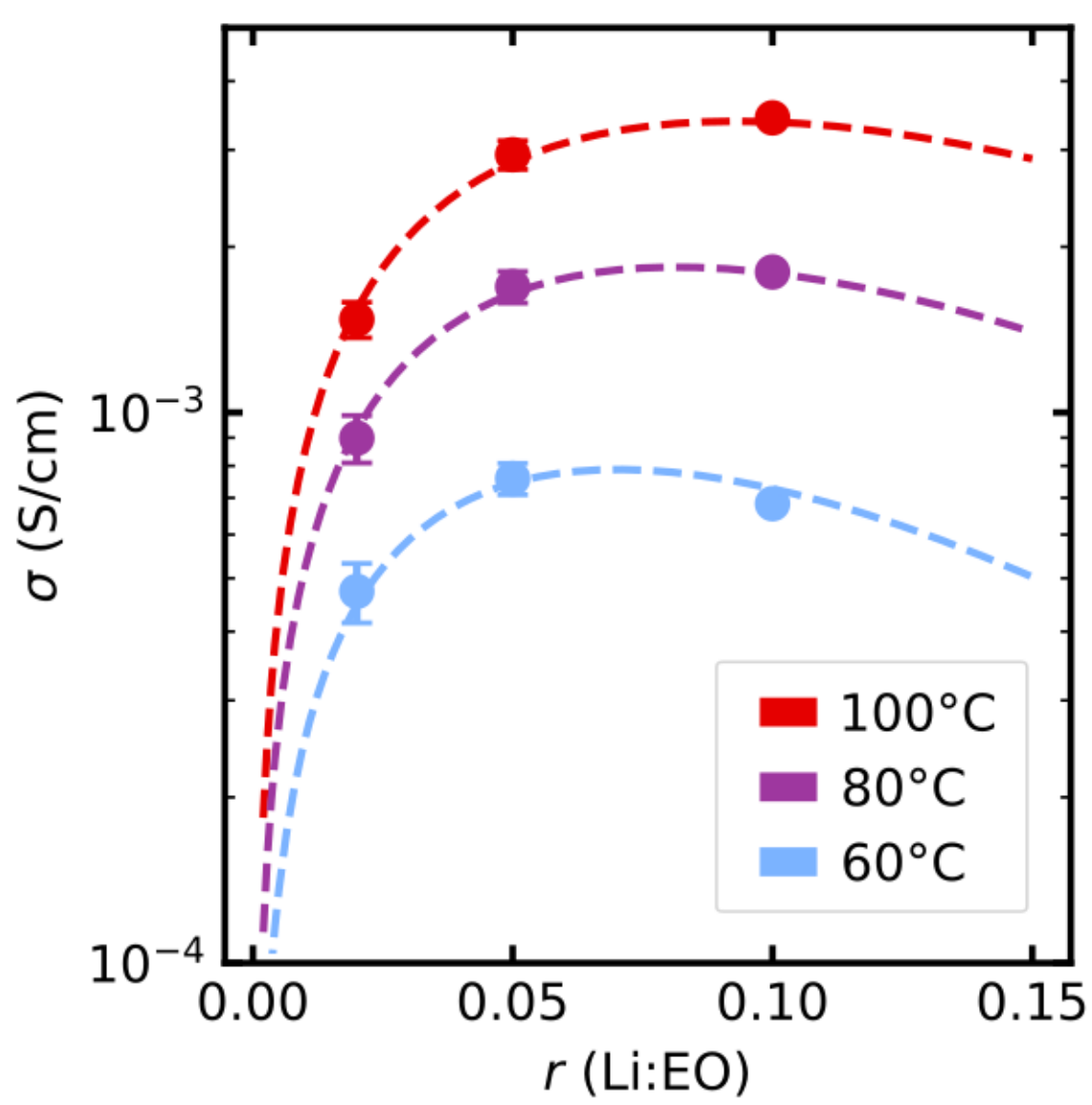

Figure S9: Bulk conductivity estimated from the bootstrap resampling procedure and their corresponding fit using an empirical model.[6] Bootstrapped results are shown as data points. Error bars are standard deviations of the bootstrapped samples but are generally smaller than marker size. Dashed lines are fits to results at each temperature using the empirical model.

S5 Experimental measurements above bulk conductivity

A number of our experimental measurements of conductivity lie above the modeled $\sigma^{(0)}$. We quantify this number by total count (Figure S10A) and their deviation from $\sigma^{(0)}$ by mean excess in percent (Figure S10B) using

$$\text{Mean Excess }\%(r,T) = \frac{\langle(\sigma(r,T) - \sigma^{(0)}(r,T))|_{\sigma>\sigma^{(0)}}\rangle}{\sigma^{(0)}(r,T)} \times 100 \tag{S16}$$

where all parameters are as previously defined.

Since the NCL, SSL, and APD models are all constrained by an implicit upper bound of $\sigma^{(0)}$, they fail to capture trends exhibited by any data that lies above $\sigma^{(0)}$. Figure S10 suggests that model failure should grow with decreased concentration, in alignment with the increase in number and deviation of measurements above $\sigma^{(0)}$. The trends in Figure S10 are negligibly dependent on temperature but suggest that model failure should be most evident at 60°C and $r = 0.02$.

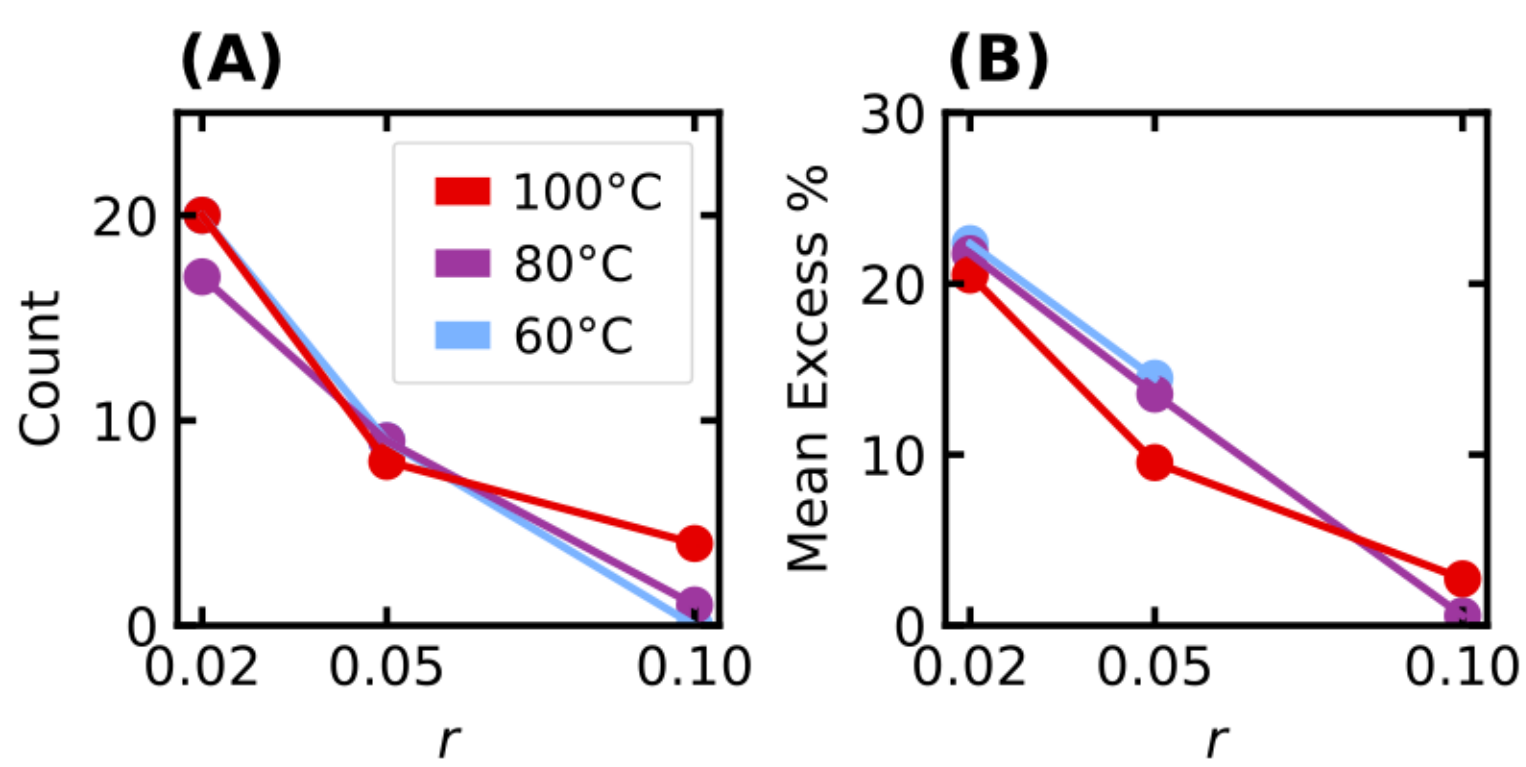

Figure S10: Experimental measurements of conductivity that exceed bulk conductivity $\sigma^{(0)}$. Measurements are quantified by (A) total number count and (B) deviation from $\sigma^{(0)}$ using mean excess percent. The legend in panel (A) applies to both panels. Solid lines are guides for the eye.

S6 Optimization of model parameters

For all models, the values of B, $E^{(0)}$, $E^{(1)}$, $T_0^{(0)}$, $a^{(1)}$, and λ retain the values optimized for bulk PEO (Section S4). Parameter optimization is done in two steps: first, nominal values are approximated using the Tree-structured Parzen Estimator (TPE) method.[10] Then, Bayesian parameter estimation (BPE) is used to refine the final parameter values and estimate uncertainties.[11]

S6.1 Initial parameter estimation using TPE

For the NCL model, z_1 is directly optimized using the Tree-structured Parzen Estimator (TPE) method[10] with regularization to penalize curvature in the parameter space. Regularization is applied using

$$L = \sum_{T} \left[m(r = 0.02, T) - 2m(r = 0.05, T) + m(r = 0.10, T)\right]^2 \tag{S17}$$

where m is a fitted parameter ($m = z_1$ for the NCL model). L is adding a penalization term to the mean-squared error (MSE) that is minimized during the TPE optimization, where MSE is defined as

$$\mathrm{MSE}(r, T) = \frac{1}{n} \sum_{\ell=1}^{n} (\log(\sigma(r, T, h_\ell)) - \log(\sigma^i(r, T, h_\ell)))^2 \tag{S18}$$

where i indicates model variation (SSL, SSLm, APD, or APD-FI), ℓ denotes an individual experimental data point, n is the total number of experimental measurements at concentration r and temperature T, and remaining parameters are as previously defined. Parameterization for all concentrations and temperatures is done simultaneously. Optimized parameters are shown in Figure S11.

The SSL model contains two parameters to be optimized, K and z_s, which we do so by holding the length scale parameter z_s constant and optimizing K using the TPE method without regularization. This is done for a range of discrete values of z_s between 0.1 and 1 independently across conditions. Regularization is not applied because it is not required to produce monotonic behavior in $a^{(2)}$ with r. At each z_s and temperature, MSE as defined in eq. S18 is summed over concentration. The value of z_s and corresponding K that minimizes this sum are taken as the optimized parameter values. This method of optimization assumes that z_s is not concentration dependent. Parameters, MSEs, and the summation of MSE over all r across all discrete values of z_s used in this process are given in Figure S12.

Optimization of parameters for the SSLm model follows a similar procedure for $z_{\mathrm{s,m}}$, K_{m}, and A as described for the SSL model, where $z_{\mathrm{s,m}}$ is the length scale constant that is held constant and K_{m} and A are optimized using the TPE method. Regularization is applied to both A and K_{m} such that two penalization terms are added to the MSE, one where $m = A$ and one where $m = K_{\mathrm{m}}$. The penalization terms are normalized by each parameter's search range squared to ensure that both terms hold similar weights. Parameters, MSEs, and the

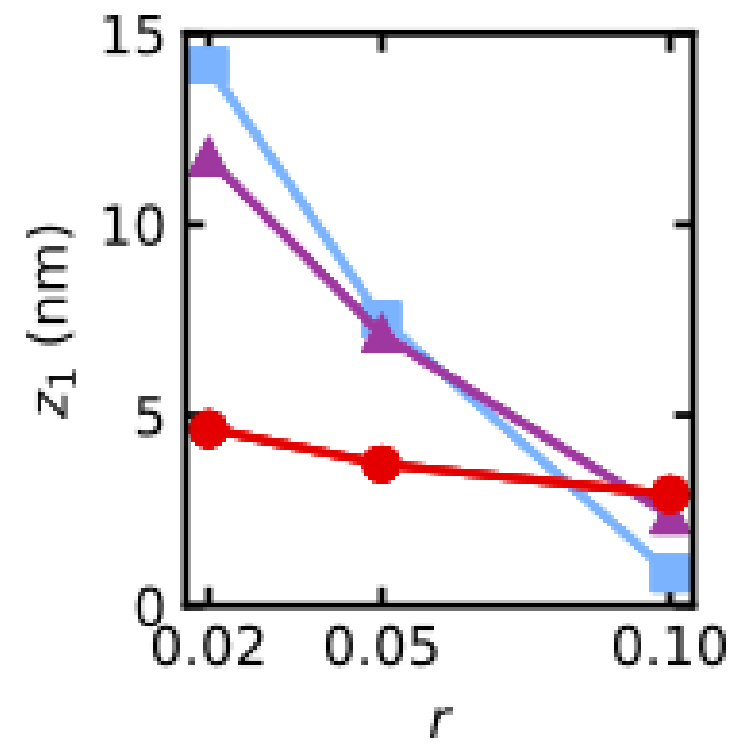

Figure S11: TPE-optimized parameters for the NCL model. Solid lines are guides for the eye.

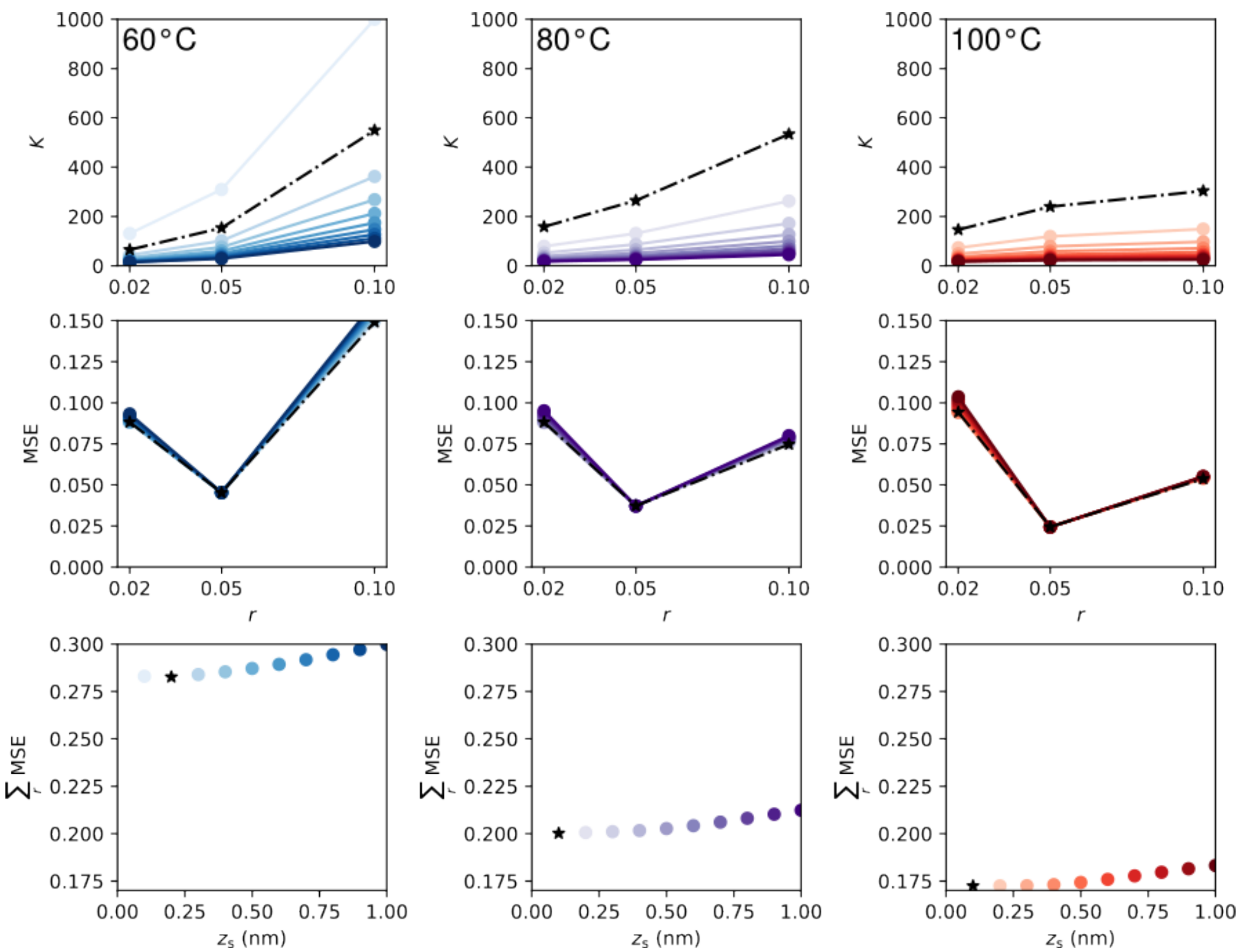

Figure S12: SSL model parameter optimization. Each column corresponds to results at one temperature (60°C, left and blue; 80°C, center and purple; 100°C, right and red). The color gradient corresponds to z_s, with lighter and darker colors indicating smaller and larger values of z_s, respectively. The plots in the bottom row show the sum of mean-squared error over r at each constant value of z_s. Results for the optimal values of z_s that minimize the sum of mean-squared error over r and corresponding values of K are shown as black stars with black dashdot lines. All lines are guides for the eye.

sum of MSE over r are shown in Figure S13.

Optimization of parameters for the APD model follows a similar procedure for $z^{(2)}$ and $a^{(2)}$ where $z^{(2)}$ is the length scale parameter that is held constant. Regularization is not applied and the parameters are optimized separately by condition. The value of $z^{(2)}$ is varied between 1 and 100. Parameters, MSEs, and the summation of MSE over all r across all discrete values of $z^{(2)}$ used in this process are given in Figure S14.

The APD-FI model contains four parameters that must be optimized: $a^{(2)}$, $z^{(2)}$, $a^{(\mathrm{f})}$, and $z^{(\mathrm{f})}$. To do so robustly, $a^{(2)}$ and $z^{(2)}$ are initially held constant at the optimized values found for the APD model. Then, $a^{(\mathrm{f})}$ and $z^{(\mathrm{f})}$ are optimized using the same method as the parameters for the SSL and APD models with $z^{(\mathrm{f})}$ varied discretely between 1 and 500. In any cases where multiple values of $z^{(\mathrm{f})}$ result in the same MSE sum over r, the smallest such $z^{(\mathrm{f})}$ is carried over. Once $a^{(\mathrm{f})}$ and $z^{(\mathrm{f})}$ are optimized, they are then held constant while $a^{(2)}$ and $z^{(2)}$ are optimized with $z^{(2)}$ varied discretely between 1 and 96. The optimized values of $a^{(2)}$ and $z^{(2)}$ from this step are then held constant while $a^{(\mathrm{f})}$ and $z^{(\mathrm{f})}$ are optimized again. This alternating optimization is repeated for 600 rounds, where optimization of either pair of parameters is considered one round. Representative parameters, MSEs, and the summation of MSE over all r across all discrete values of $z^{(\mathrm{f})}$ tested for round 599 and $z^{(2)}$ tested for round 600 are shown in Figures S15 and S16, respectively. Figure S17 shows how the parameters and summation of MSE over r evolve over the optimization rounds. Regularization is not applied in this optimization procedure.

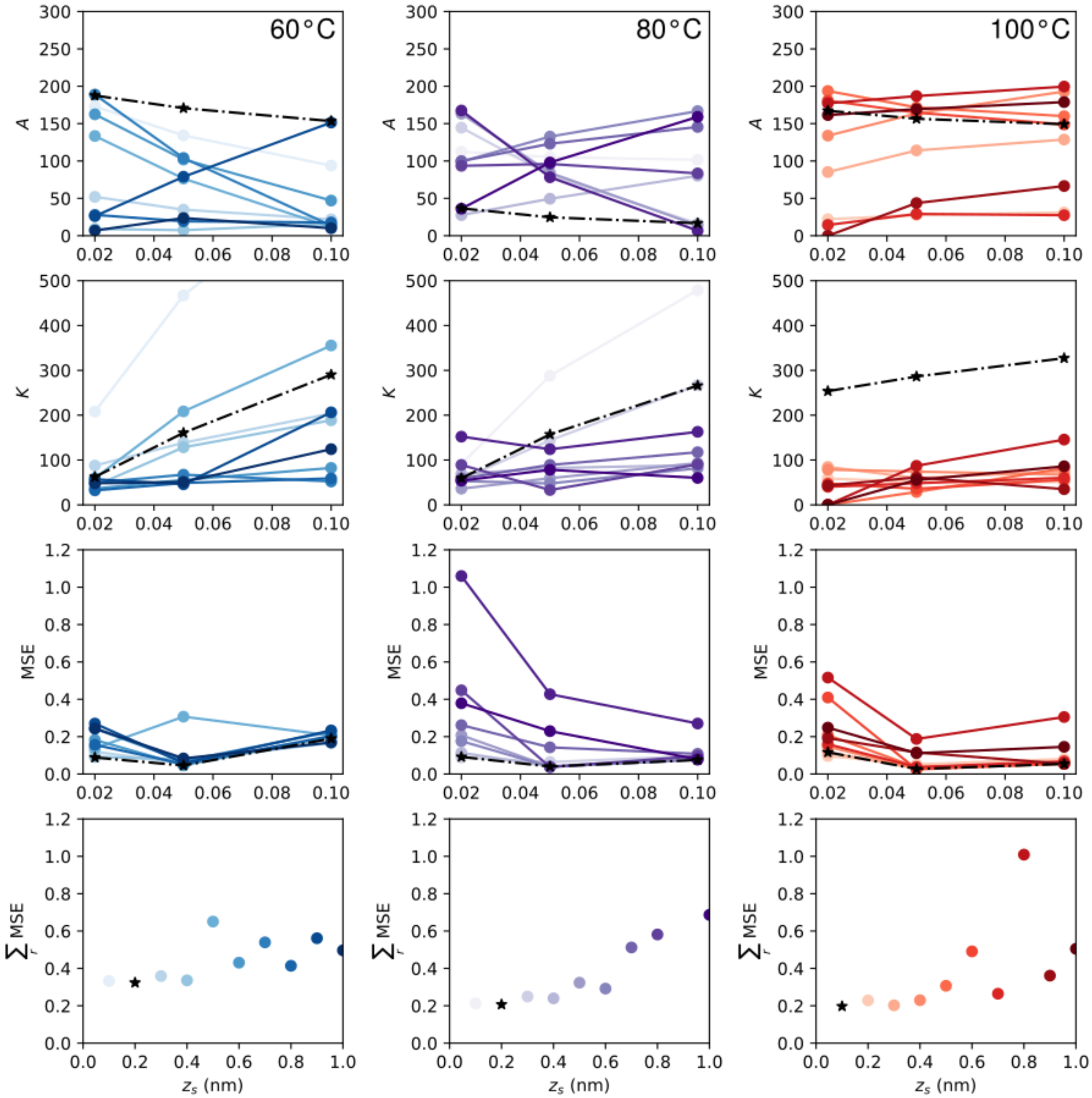

Figure S13: SSLm model parameter optimization. Each column corresponds to results at one temperature (60°C, left and blue; 80°C, center and purple; 100°C, right and red). The color gradient corresponds to $z_{\mathrm{s,m}}$, with lighter and darker colors indicating smaller and larger values of $z_{\mathrm{s,m}}$, respectively. The plots in the bottom row show the sum of mean-squared error over r at each constant value of $z_{\mathrm{s,m}}$. Results for the optimal values of $z_{\mathrm{s,m}}$ that minimize the sum of mean-squared error over r and corresponding values of K_{m} and A are shown as black stars with black dashdot lines. All lines are guides for the eye.

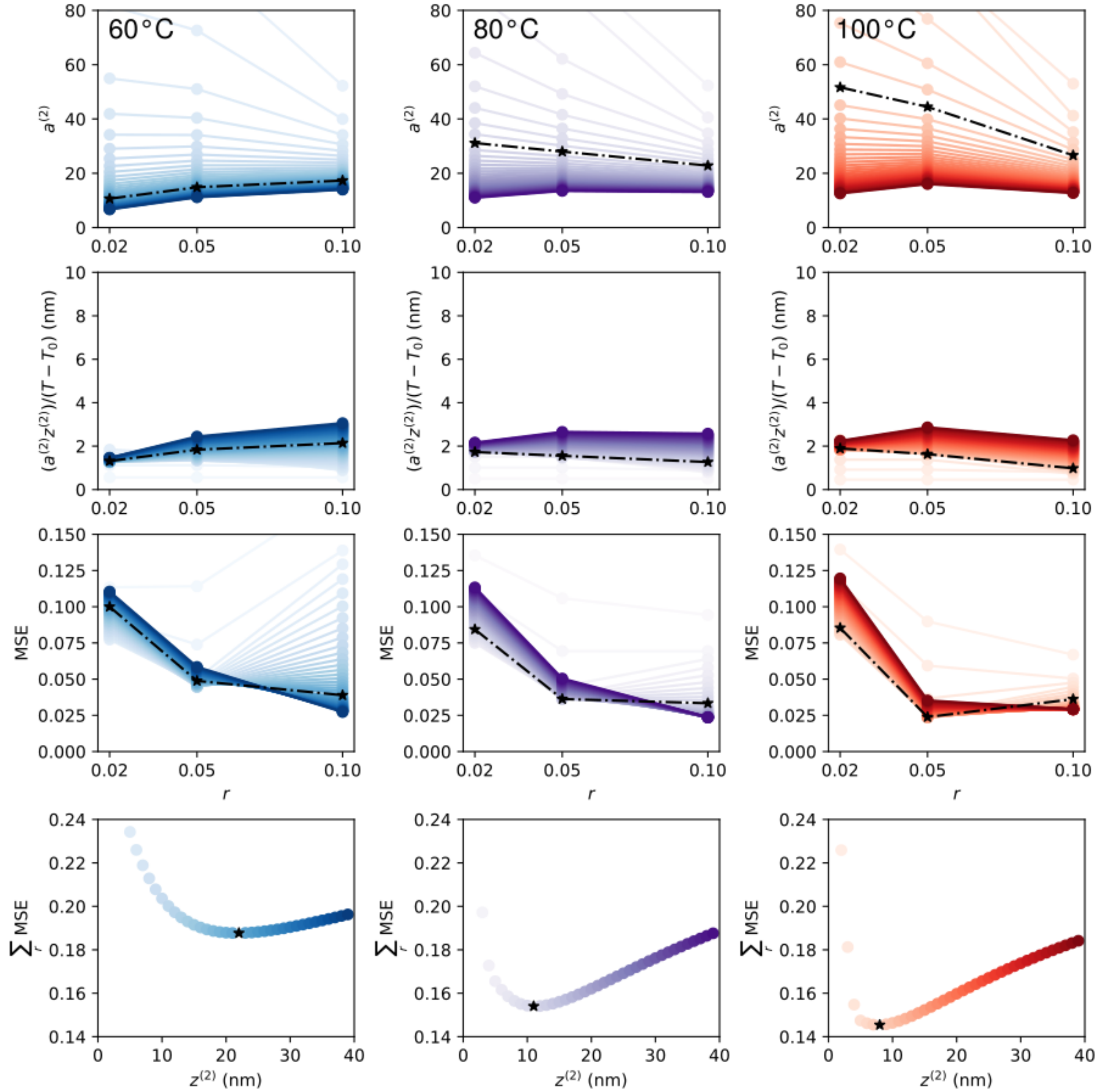

Figure S14: APD model parameter optimization. Each column corresponds to results at one temperature (60°C, left and blue; 80°C, center and purple; 100°C, right and red). The color gradient corresponds to $z^{(2)}$, with lighter and darker colors indicating smaller and larger values of $z^{(2)}$, respectively. The plots in the bottom row show the sum of mean-squared error over r at each constant value of $z^{(2)}$. Results for the optimal values of $z^{(2)}$ that minimize the sum of mean-squared error over r and corresponding values of $a^{(2)}$ are shown as black stars with black dashdot lines. All lines are guides for the eye.

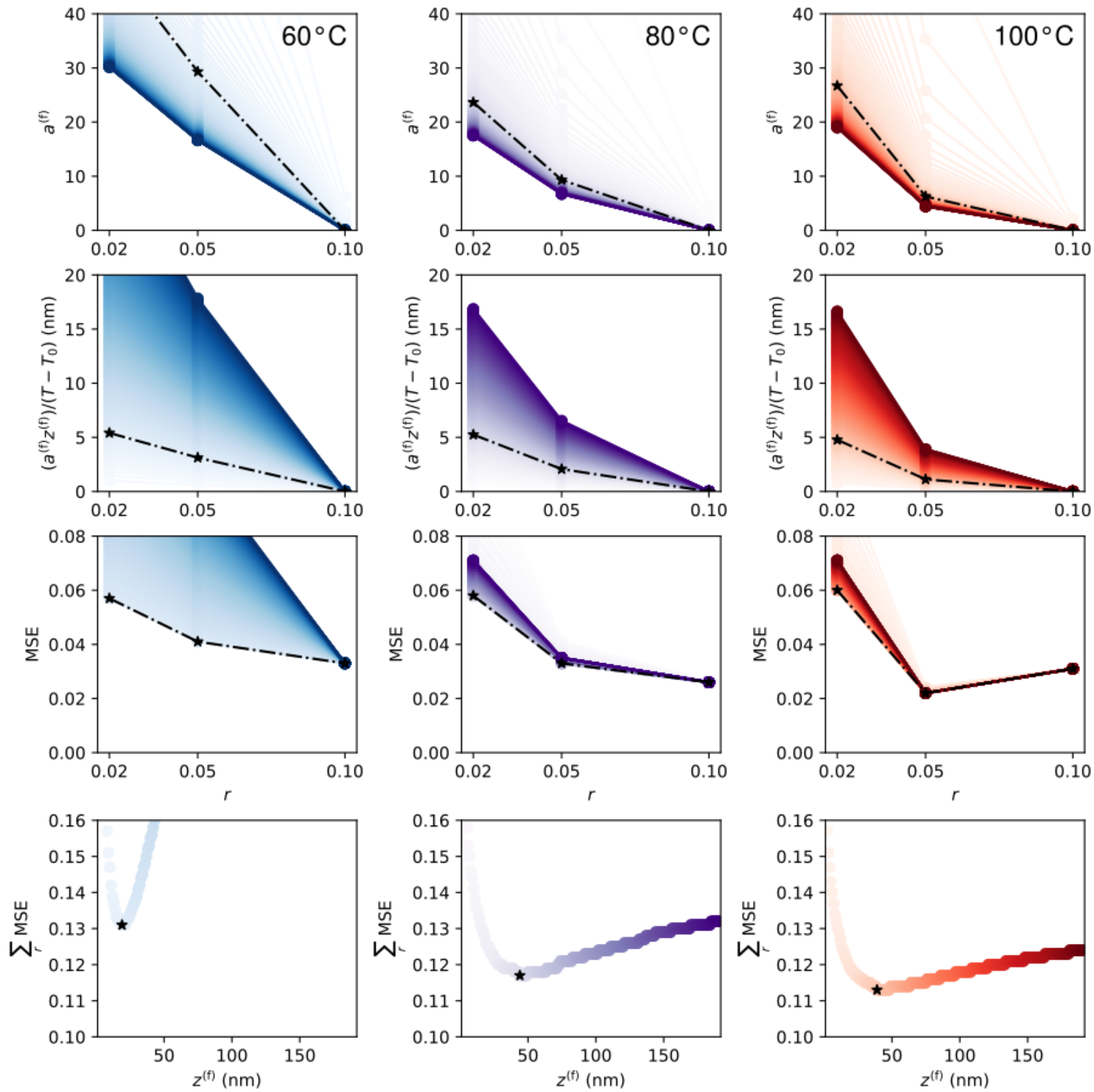

Figure S15: Parameter optimization of $z^{(f)}$ and $a^{(f)}$ for the APD-FI model. Results are for round 599 of the optimization process. Each column corresponds to results at one temperature (60°C, left and blue; 80°C, center and purple; 100°C, right and red). The color gradient corresponds to $z^{(f)}$, with lighter and darker colors indicating smaller and larger values of $z^{(f)}$, respectively. The plots in the bottom row show the sum of mean-squared error over r at each constant value of $z^{(f)}$. Results for the optimal values of $z^{(f)}$ that minimize the sum of mean-squared error over r and corresponding values of $a^{(f)}$ are shown as black stars with black dashdot lines. All lines are guides for the eye.

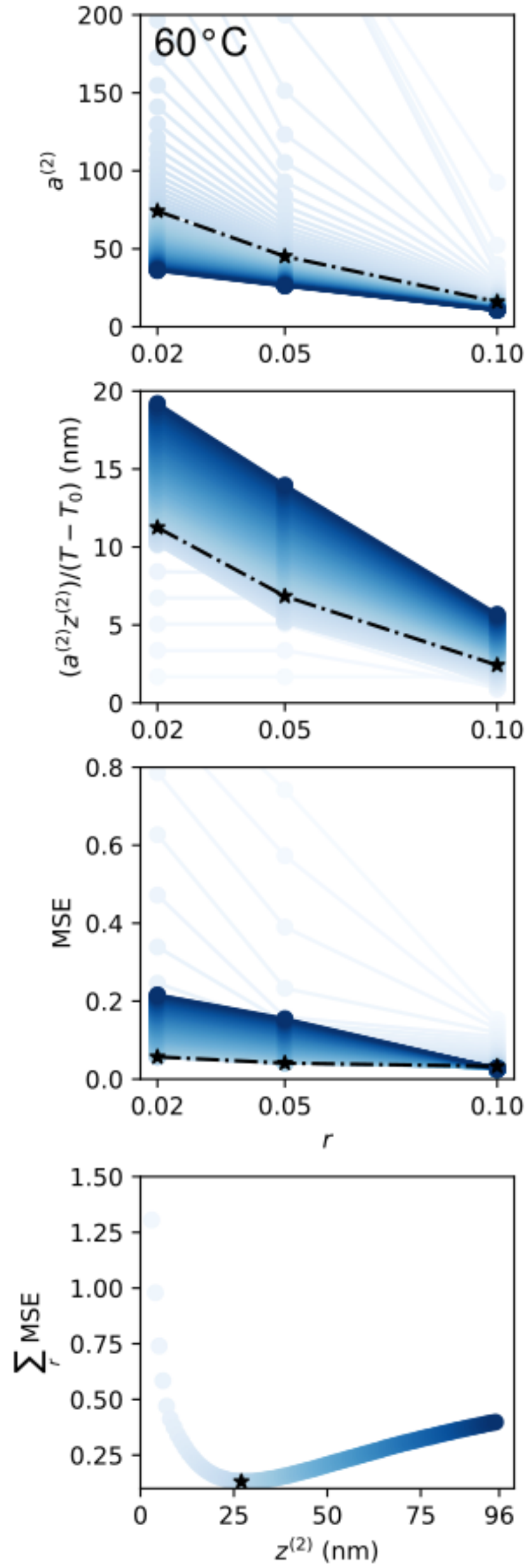

Figure S16: Parameter optimization of $z^{(2)}$ and $a^{(2)}$ for the APD-FI model. Results are for round 600 of the optimization process. Each column corresponds to results at one temperature (60°C, left and blue; 80°C, center and purple; 100°C, right and red). The color gradient corresponds to $z^{(2)}$, with lighter and darker colors indicating smaller and larger values of $z^{(2)}$, respectively. The plots in the bottom row show the sum of mean-squared error over r at each constant value of $z^{(2)}$. Results for the optimal values of $z^{(2)}$ that minimize the sum of mean-squared error over r and corresponding values of $a^{(2)}$ are shown as black stars with black dashdot lines. All lines are guides for the eye.

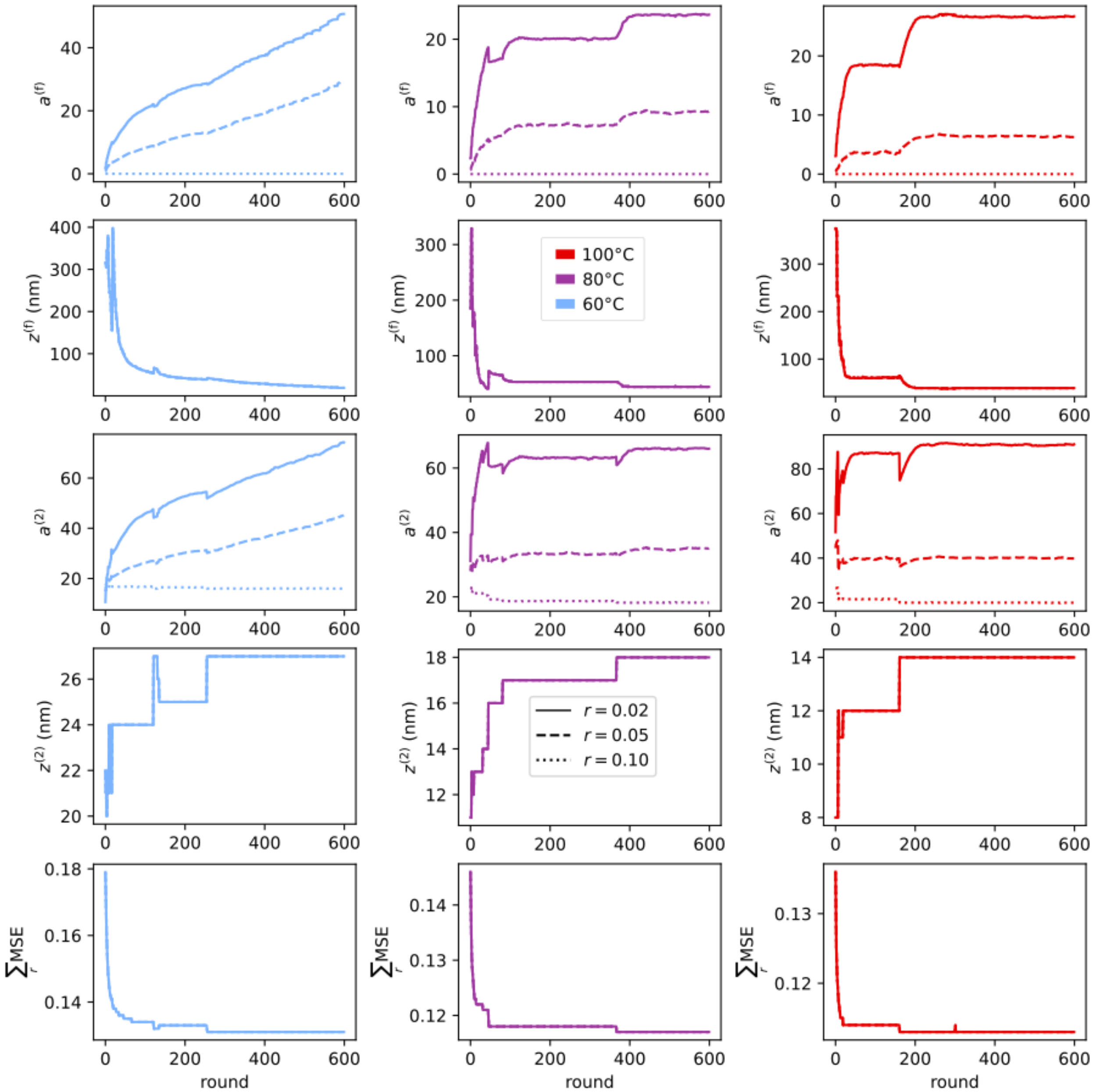

Figure S17: Evolution of APD-FI model parameters and mean-squared error summed over r over 600 rounds of optimization. The legends in the second and third rows of the center column indicating data at different concentrations and temperatures applies to all panels. Figures showing only solid lines contain overlaid results from all three concentrations.

S6.2 Parameter refinement and uncertainty estimation using BPE

For each model, the nominal parameter values from the TPE method are used as the central values for prior distributions in BPE. Prior distributions are all truncated Normal. The standard deviations of the priors are varied to achieve converged posterior sampling. Truncation ranges and standard deviations for each parameter in each model are given in Table S3. The likelihood for each data point is assumed to follow a Normal distribution with a fixed standard deviation calculated from the five conductivity measurements with the nearest h-values at the same r and T, including the selected data point itself. Figure S18 shows these estimated standard deviations for all conductivity measurements. Figure S19 shows the standard deviations of h across these same measurements.

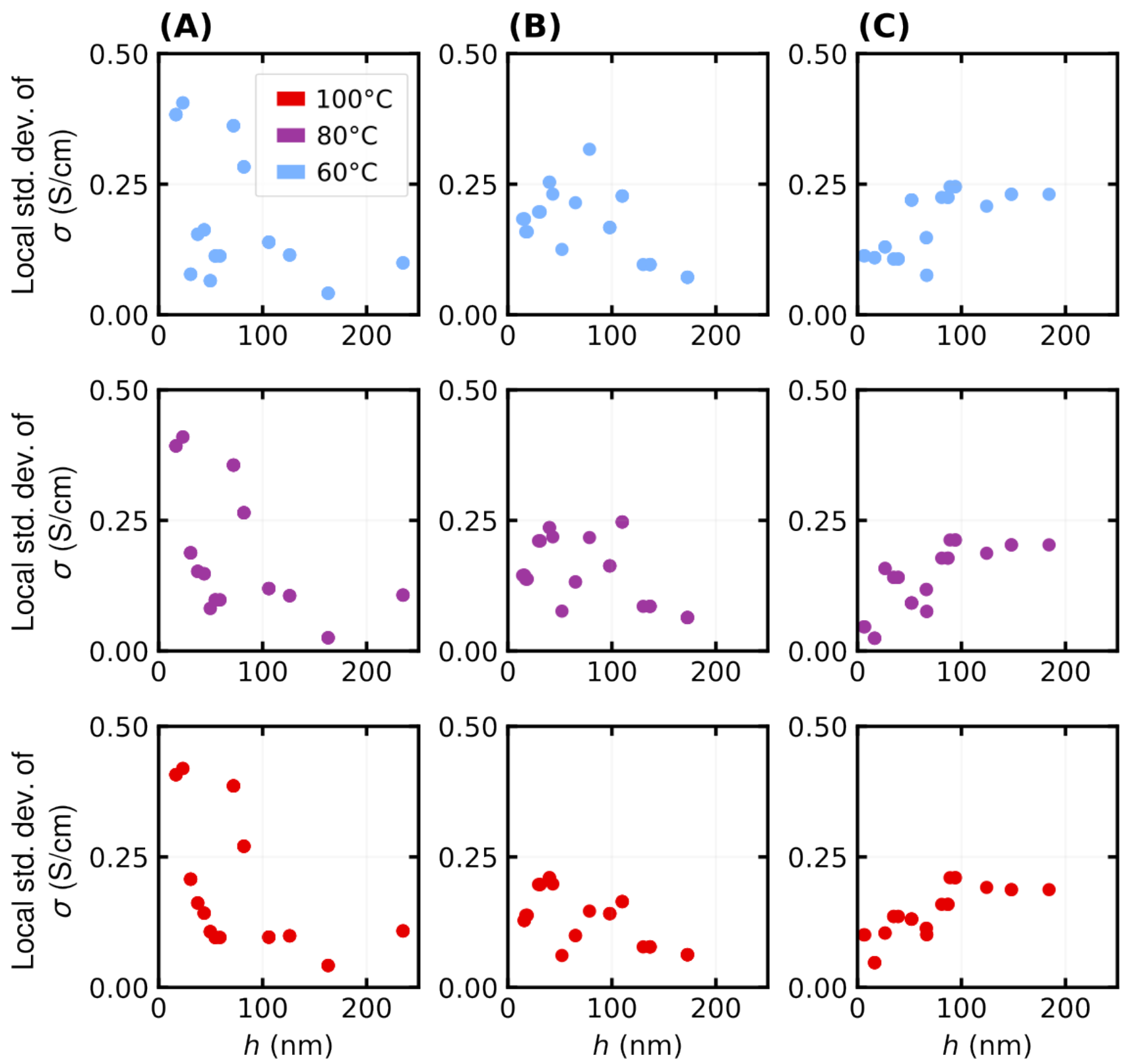

Figure S18: Conductivity variability across the experimental dataset calculated as the standard deviation of σ across a given point and the four measurements with the nearest h values at the same r and T.

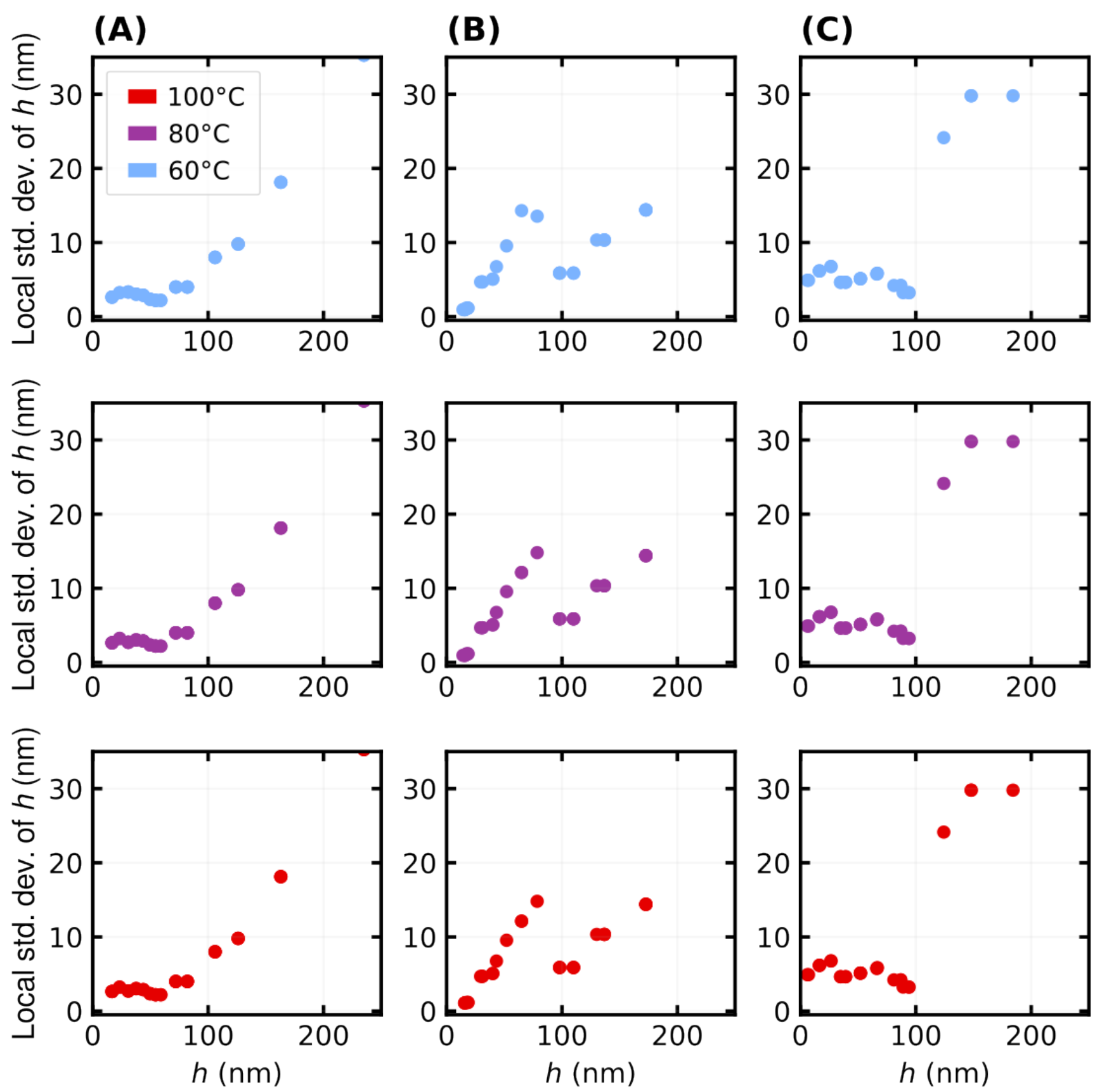

Figure S19: Film thickness variability across the experimental dataset calculated as the standard deviation of h across a given point and the four measurements with the closest matching h values at the same r and T.

Table S3: Standard deviations and truncation ranges for truncated Normal prior distributions used during Bayesian parameter estimation.

Model	Parameter	Standard Deviation	Truncation Range
SSL	K	10	[0, 1000]
SSL	z_{s}	1	[0.1, 20]
SSLm	K_{m}	20	[0, 1000]
SSLm	$z_{\mathrm{s,m}}$	1	[0.1, 20]
SSLm	A	20	[0, 1000]
NCL	z_1	1	[0, 20]
APD	$z^{(2)}$	10	[1, 200]
APD	$a^{(2)}$	10	[1, 200]
APD-FI	$z^{(2)}$	10	[1, 200]
APD-FI	$a^{(2)}$	10	[1, 200]
APD-FI	$z^{(\mathrm{f})}$	10	[1, 200]
APD-FI	$a^{(\mathrm{f})}$	10	[1, 200]

Posterior sampling is done using the No-U-Turn Sampler (NUTS) and four independent chains. Convergence is determined by asserting that the Gelman-Rubin statistic ($\hat{r}$) is less than 1.01 for all parameters. For the APD and APD-FI models, we assess the robustness of NUTS sampling for the coupled $a^{(2)}/z^{(2)}$ and $a^{(\mathrm{f})}/z^{(\mathrm{f})}$ parameters to initial conditions by running four chains from distinct starting points: the TPE-derived values, minimum $a^{(2)}$/maximum $z^{(2)}$ and minimum $a^{(\mathrm{f})}$/maximum $z^{(\mathrm{f})}$ values, maximum $a^{(2)}$/minimum $z^{(2)}$ and maximum $a^{(\mathrm{f})}$/minimum $z^{(\mathrm{f})}$ values, and a random draw from the prior. Joint posterior distributions colored by chain for these two models are provided in Figures S20-S22. At every condition and for both models, the different chains all explore the parameter space similarly and lead to converged posterior means.

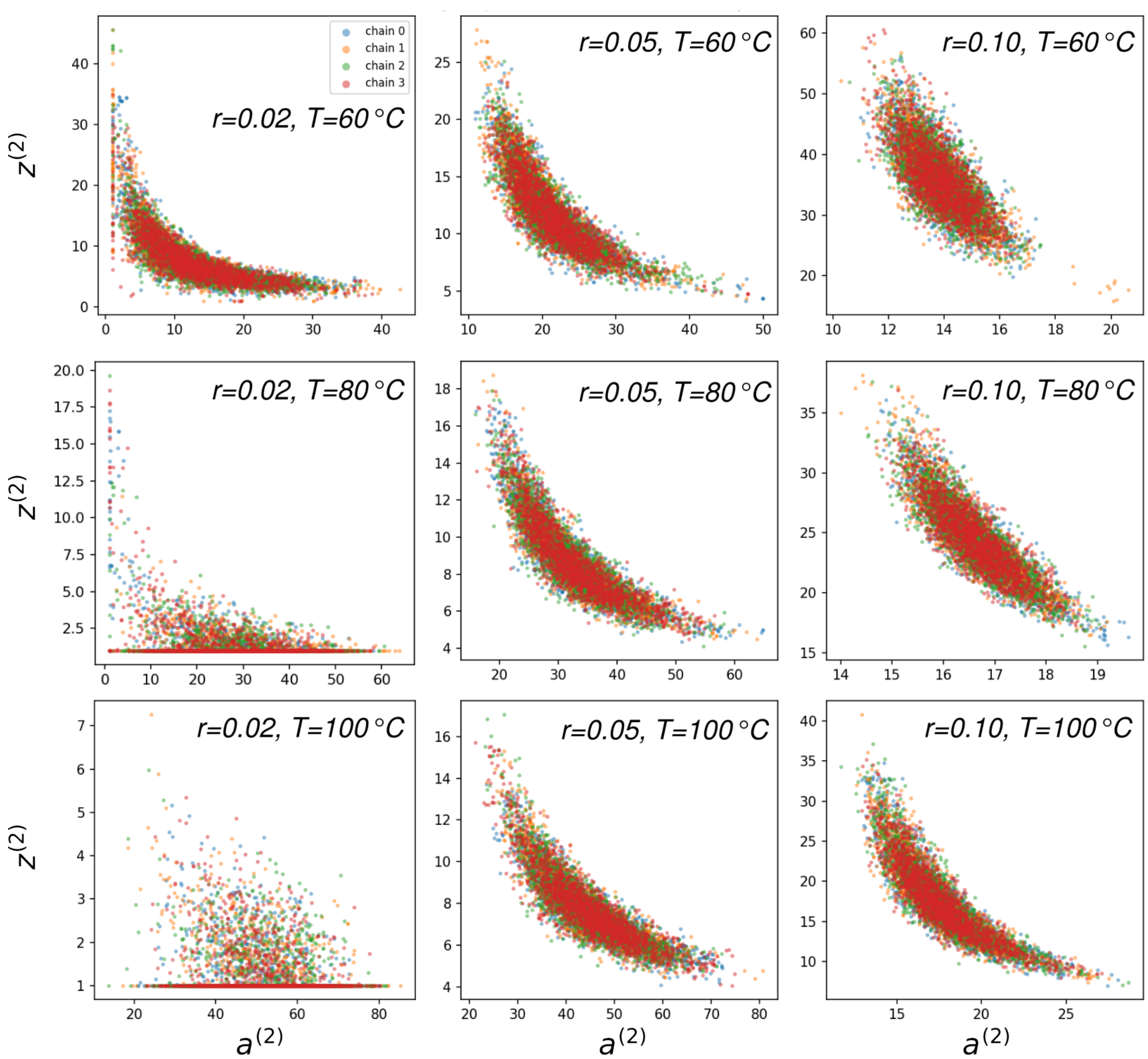

Figure S20: Convergence of joint posterior distributions of $a^{(2)}$ and $z^{(2)}$ for the APD model across four independently initialized NUTS chains. The legend in the top left panel indicating chain applies for all panels.

Comparison of model predictions to experimental data following TPE optimization and BPE optimization are given for the NCL, SSL, SSLm, APD, and APD-FI models in Figures S23-S27.

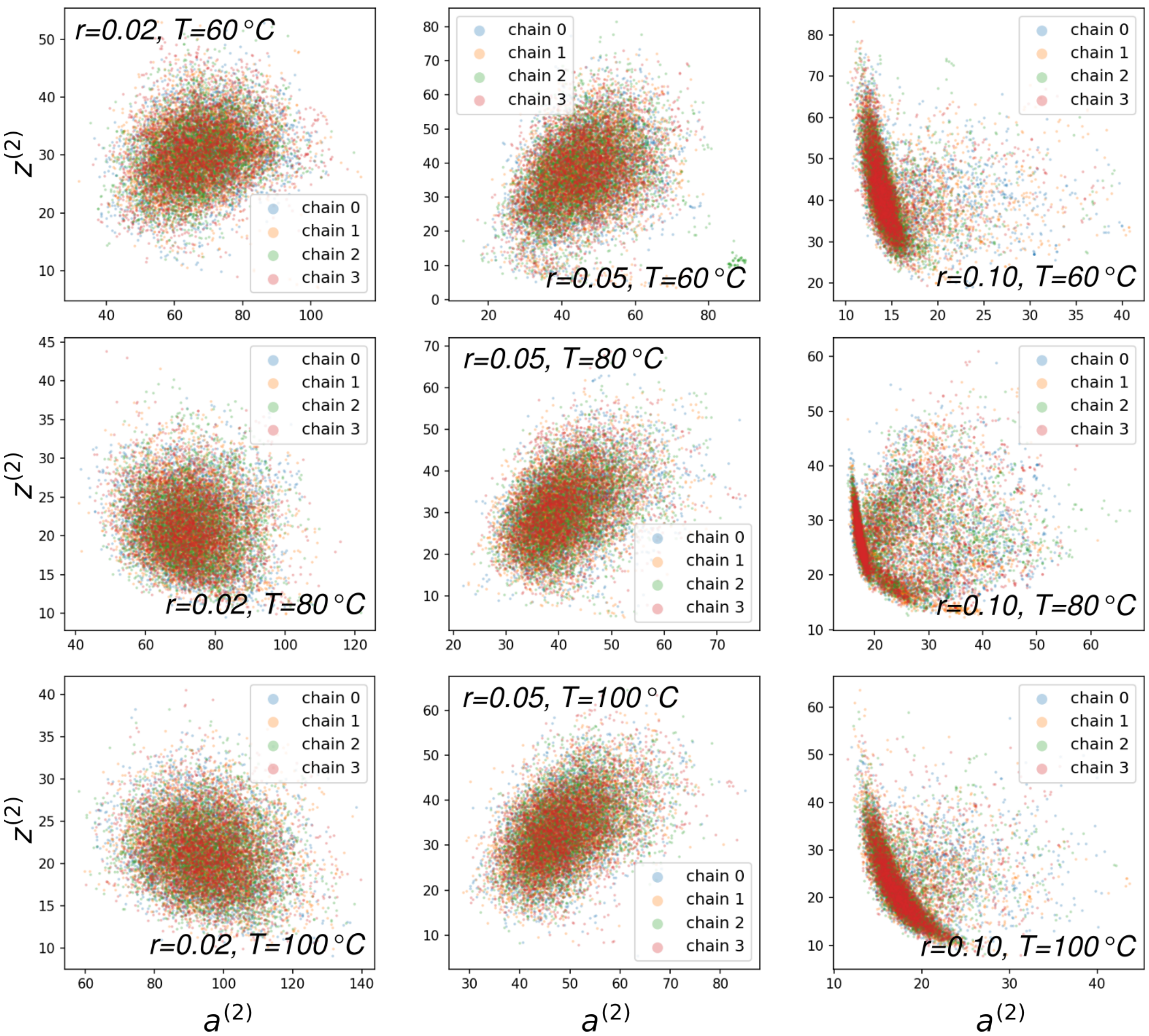

Figure S21: Convergence of joint posterior distributions of $a^{(2)}$ and $z^{(2)}$ for the APD-FI model across four independently initialized NUTS chains.

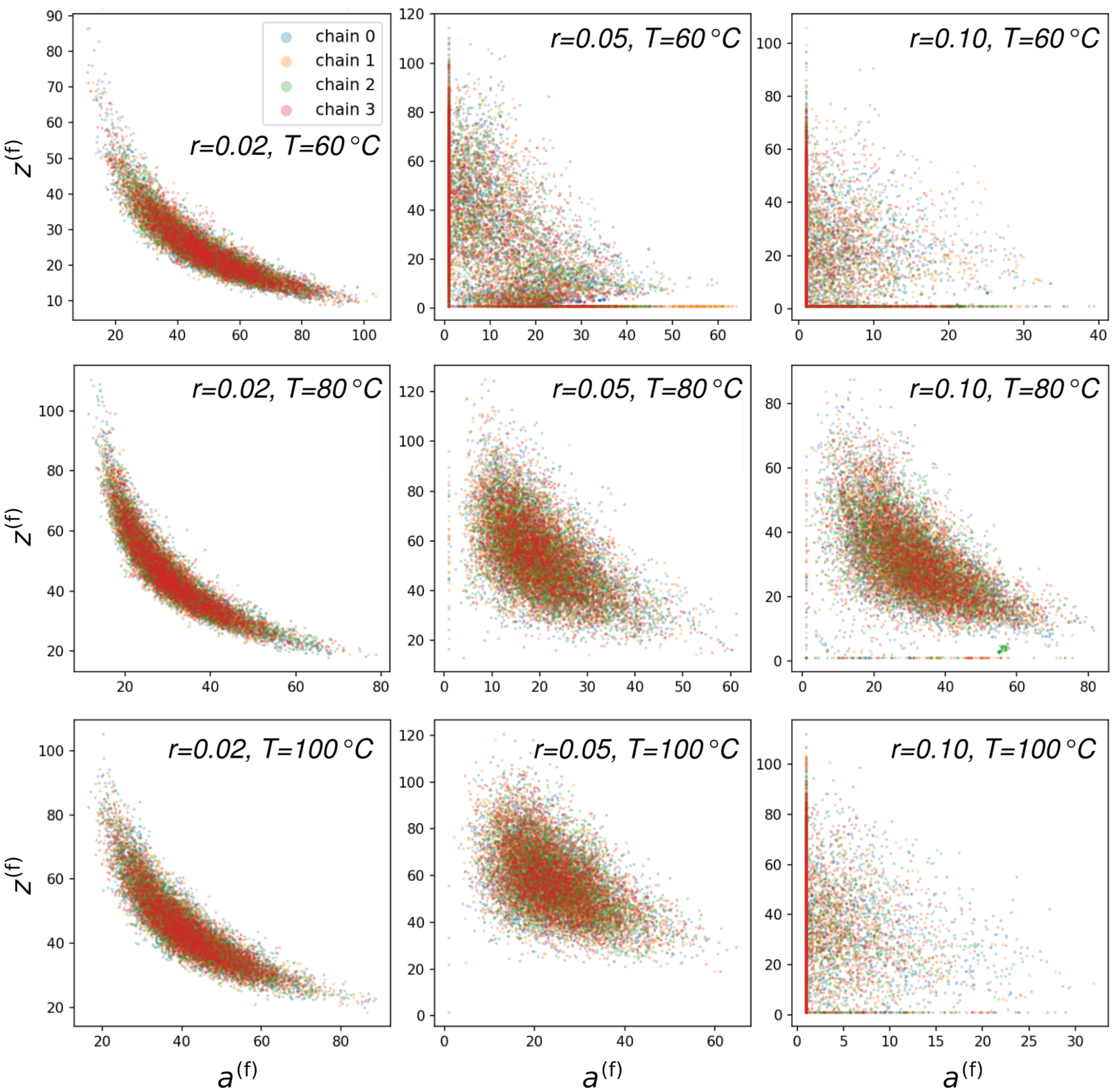

Figure S22: Convergence of joint posterior distributions of $a^{(\mathrm{f})}$ and $z^{(\mathrm{f})}$ for the APD-FI model across four independently initialized NUTS chains. The legend in the top left panel indicating chain applies for all panels.

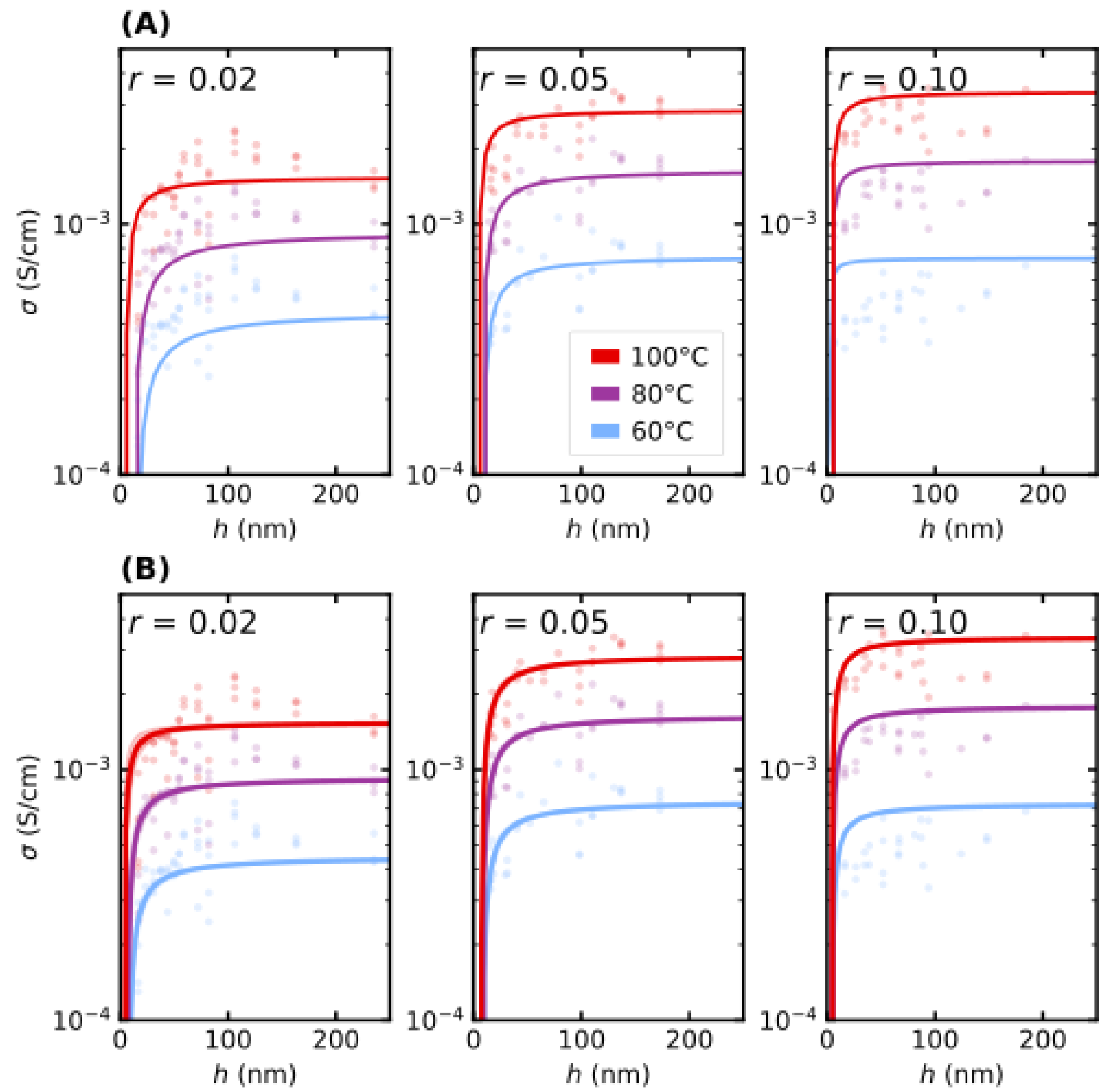

Figure S23: NCL model predictions following (A) TPE optimization and (B) BPE optimization. Transparent points are experimental data points. Solid lines are model results using optimized parameter values. For BPE optimization, the solid lines are results from median values of the optimized parameter posteriors and shaded regions are 50% (light) and 95% (dark) credible intervals. The legend in the center panel of (A) applies to all panels.

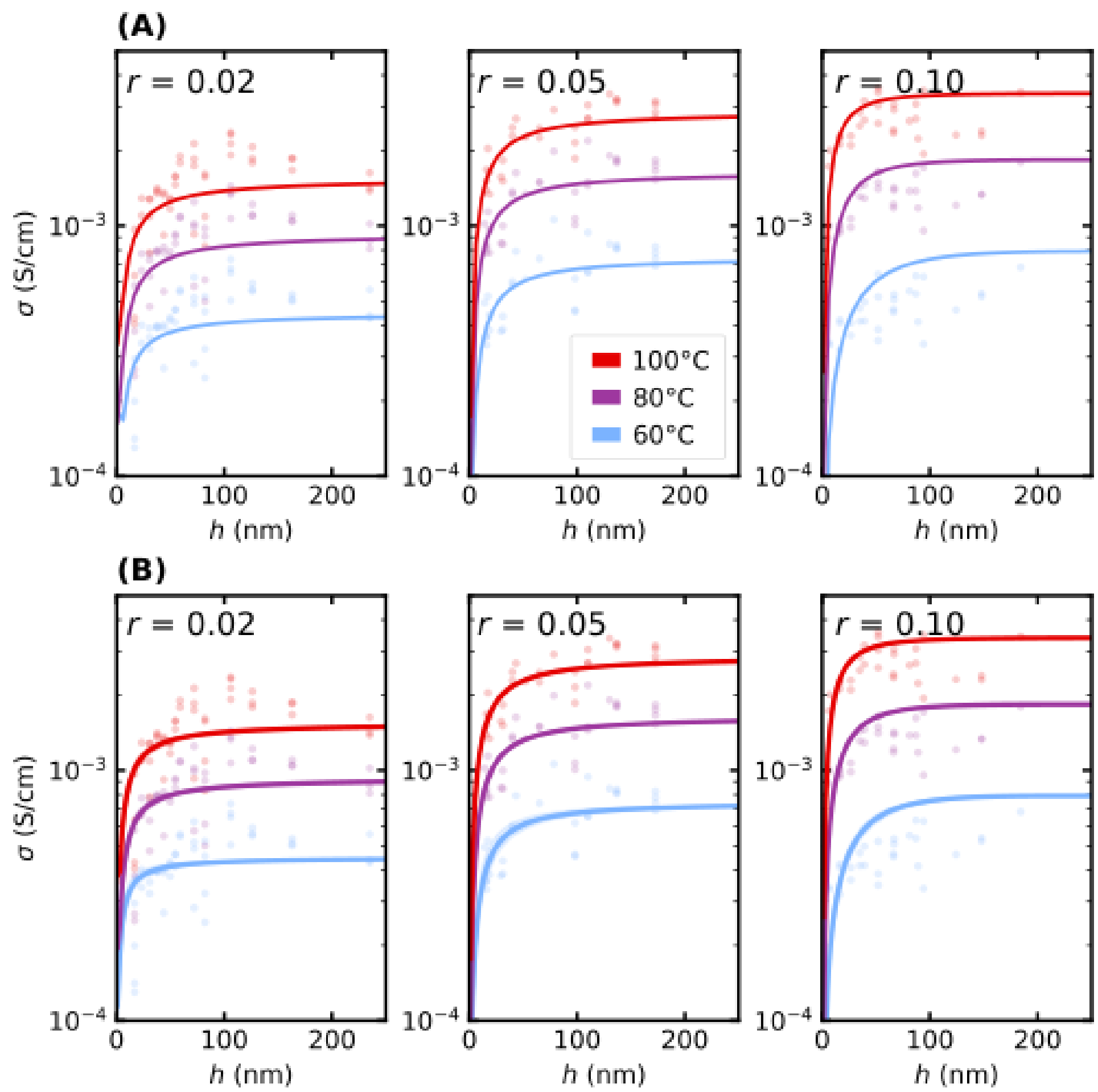

Figure S24: SSL model predictions following (A) TPE optimization and (B) BPE optimization. Transparent points are experimental data points. Solid lines are model results using optimized parameter values. For BPE optimization, the solid lines are results from median values of the optimized parameter posteriors and shaded regions are 50% (light) and 95% (dark) credible intervals. The legend in the center panel of (A) applies to all panels.

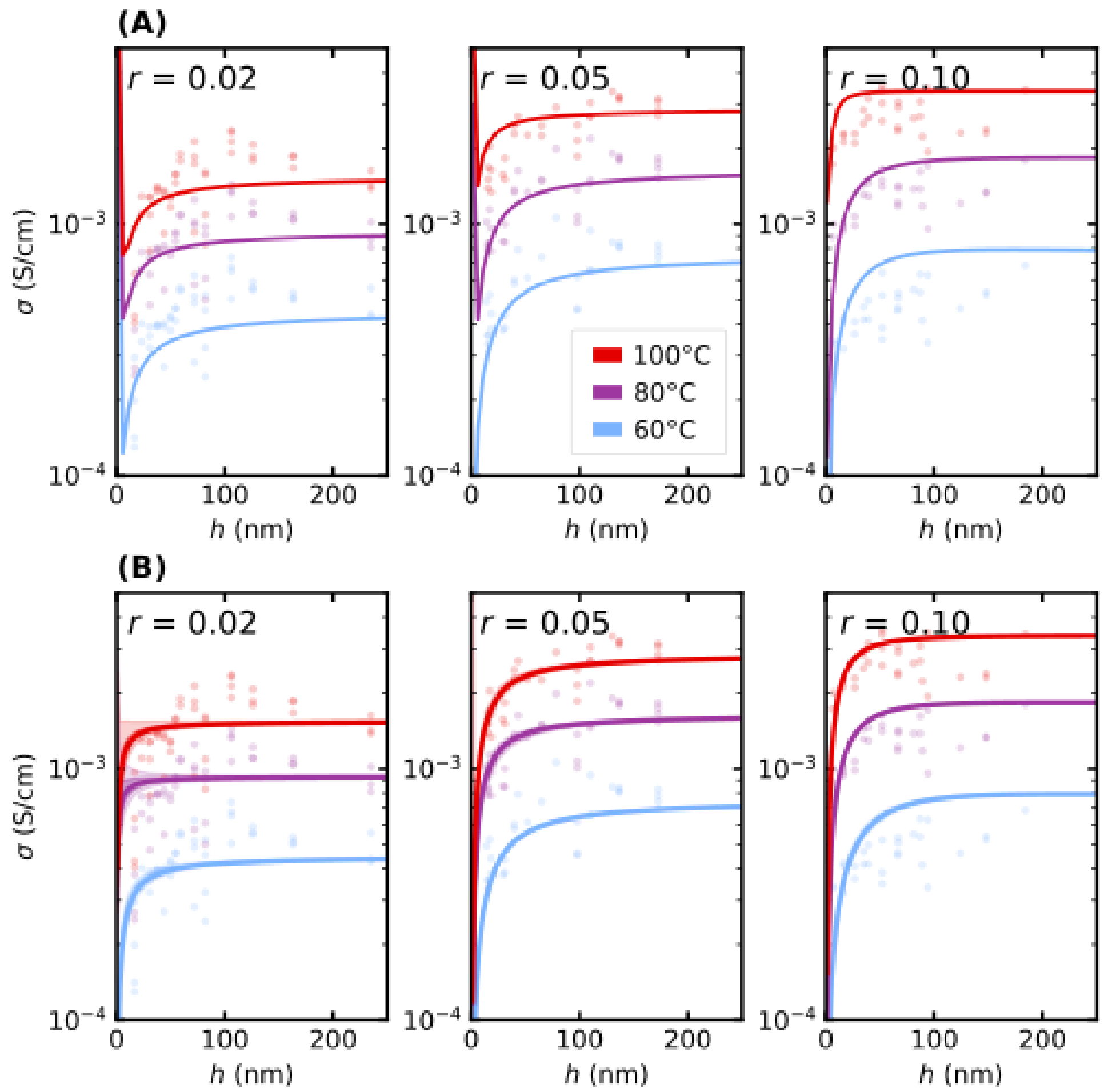

Figure S25: SSLm model predictions following (A) TPE optimization and (B) BPE optimization. Transparent points are experimental data points. Solid lines are model results using optimized parameter values. For BPE optimization, the solid lines are results from median values of the optimized parameter posteriors and shaded regions are 50% (light) and 95% (dark) credible intervals. The legend in the center panel of (A) applies to all panels.

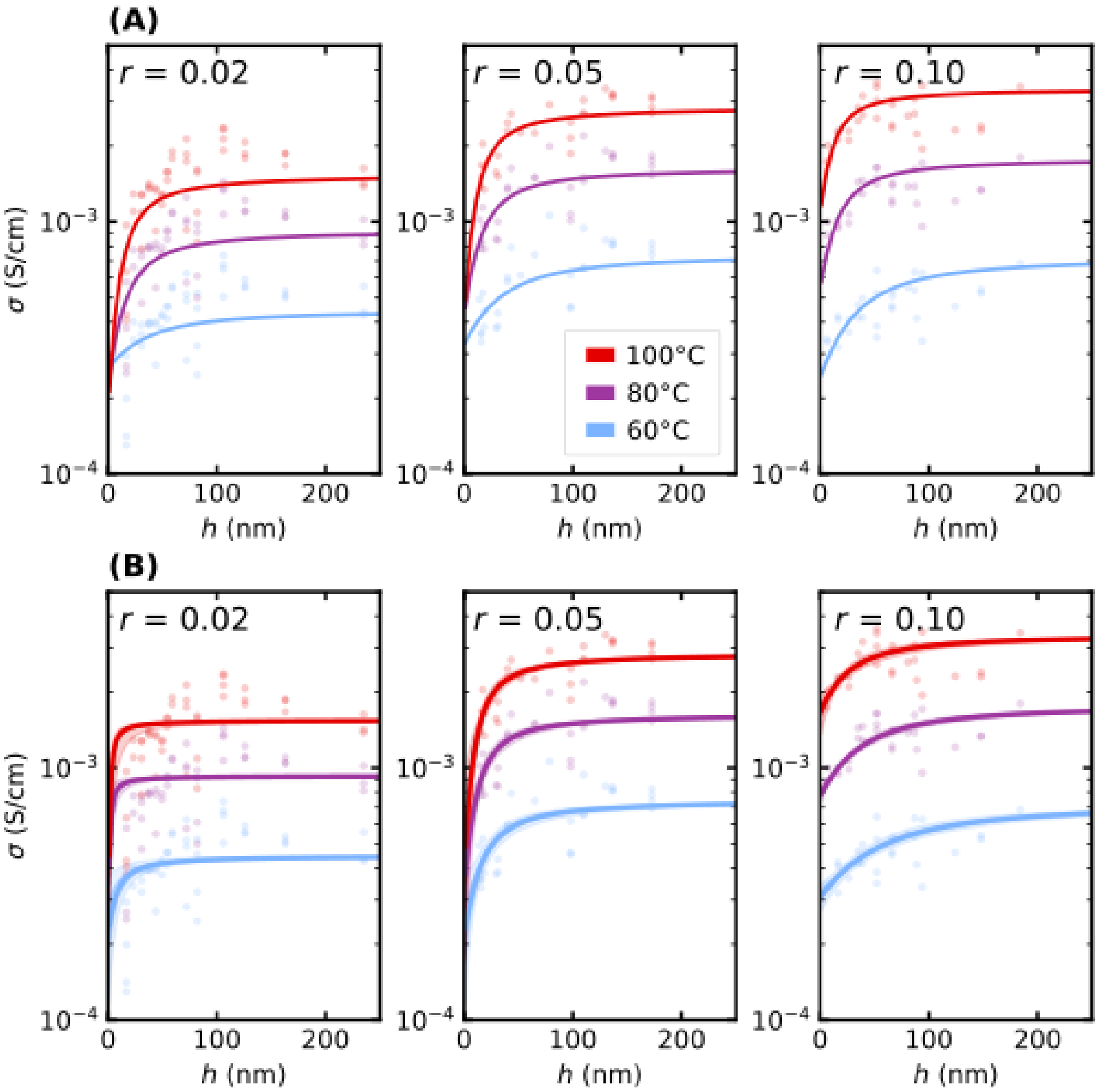

Figure S26: APD model predictions following (A) TPE optimization and (B) BPE optimization. Transparent points are experimental data points. Solid lines are model results using optimized parameter values. For BPE optimization, the solid lines are results from median values of the optimized parameter posteriors and shaded regions are 50% (light) and 95% (dark) credible intervals. The legend in the center panel of (A) applies to all panels.

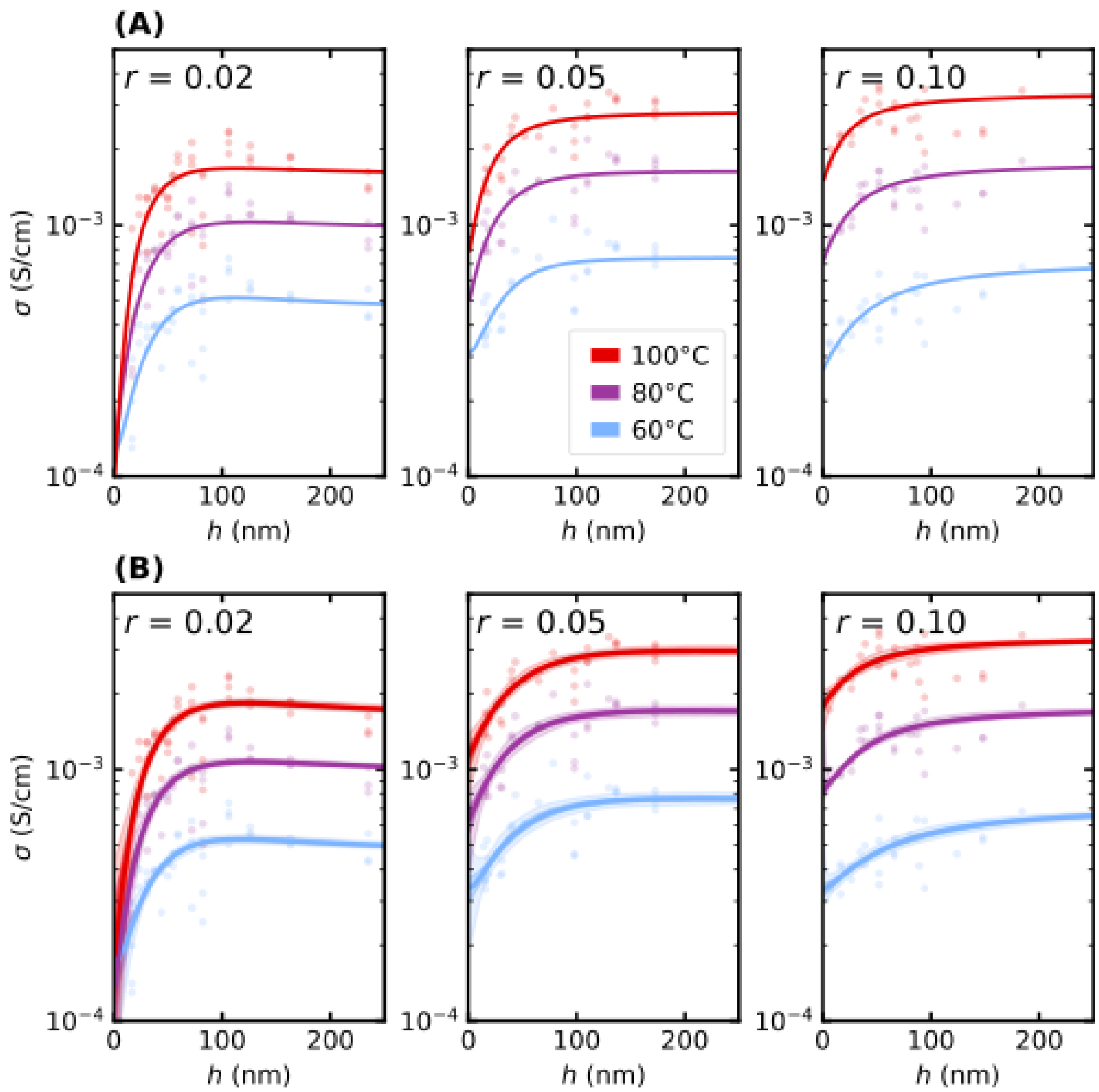

Figure S27: APD-FI model predictions following (A) TPE optimization and (B) BPE optimization. Transparent points are experimental data points. Solid lines are model results using optimized parameter values. For BPE optimization, the solid lines are results from median values of the optimized parameter posteriors and shaded regions are 50% (light) and 95% (dark) credible intervals. The legend in the center panel of (A) applies to all panels.

S7 Model Comparison

We compare models using ELPD on log-transformed conductivities and the coefficient of determination (R^2) on non-transformed conductivities. The ELPD calculation is done using leave-one-out cross-validation (LOO-CV). For each model and salt concentration/temperature condition, a posterior distribution over model parameters is obtained (9 conditions $\times$ 5 models = 45 total posterior distributions). The ELPD is then calculated for each posterior by summing the pointwise expected log predictive densities over the experimental measurements. Each posterior is evaluated against the complete experimental dataset across all salt concentrations and temperatures. The resulting ELPD values, which are stratified by model and condition, are then summed over all conditions to obtain the final model-level ELPD values reported in Figure 5A.

In Figure S28, we also provide ELPD values of the APD and APD-FI models stratified by film thickness. Although Figure 5A shows statistically similar model-level ELPD values for APD and APD-FI, Figure S28 shows that the marginal improvement of APD-FI is driven almost entirely by films with $r = 0.02$. At this salt concentration, the APD-FI model outperforms the APD model at $h \in [0, 40]$ nm and $h \in [100, 120]$ nm. This is due to to the added flexibility of the competing interfacial effects in the APD-FI. The free interface term allows conductivity to exceed bulk, leading to better performance at $h \in [100, 120]$ nm, while the substrate term captures the steep drop-off in conductivity at small h that is unachievable by the APD model. However, the APD model performs better at $h \in [60, 100]$ nm due to the large sample-to-sample variability in this regime (Figure S2), which is better described by the bulk prediction of the APD model rather than the enhanced conductivity of the APD-FI model. Given these results, interpretation of the conductivity enhancement is uncertain and warrants future study with reduced experimental variability.

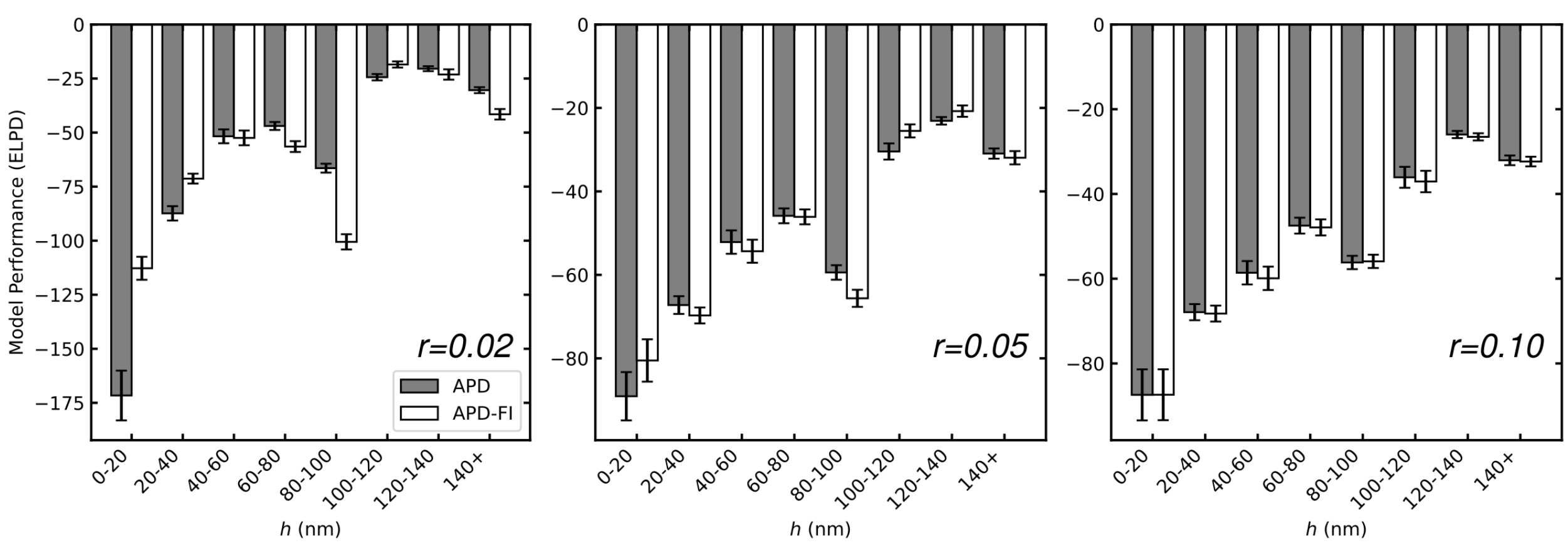

Figure S28: Model performance of the APD and APD-FI models stratified by bands of film thickness. Error bars represent the standard error calculated over all experimental measurements of conductivity in the h band. The legend in the left panel indicating model type applies to all panels.

Residual plots and coefficient of determination values (R^2) of each model at each condition are shown in Figure S29. The lowest R^2 values occur at $r = 0.10$ across all temperatures. We attribute this to the reduced interfacial effect with increasing r (quantified using ξ in the

main text). As the effect becomes weaker, the model fit becomes both worse and increasingly sensitive to experimental sample-to-sample scatter. For a direct comparison of each model across all conditions, we sum up the R^2 values at each condition for final comparison values of 3.98, 2.78, 2.16, -2.31, and -2.57 for the APD-FI, APD, SSL, NCL, and SSLm models, respectively.

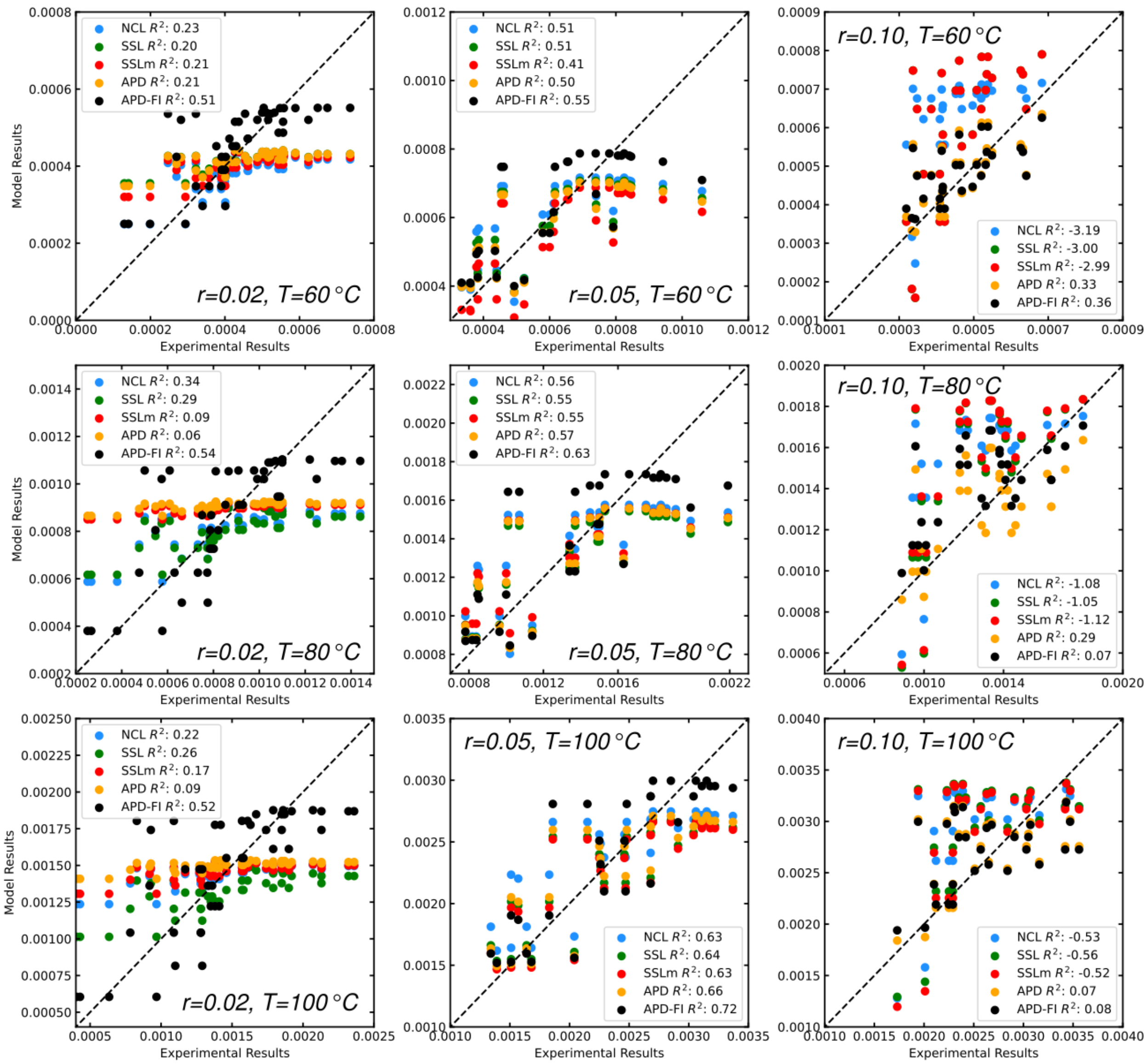

Figure S29: Residual plots of experimental data versus model results and corresponding R^2 values. The dashed black lines are guides for the eye.

References

(1) Sharon, D.; Bennington, P.; Liu, C.; Kambe, Y.; Dong, B. X.; Burnett, V. F.; Dolejsi, M.; Grocke, G.; Patel, S. N.; Nealey, P. F. Interrogation of Electrochemical Properties of Polymer Electrolyte Thin Films with Interdigitated Electrodes. *Journal of The Electrochemical Society* **2018**, *165*, H1028, DOI: 10.1149/2.0291816jes.

(2) Marzantowicz, M.; Dygas, J. R.; Krok, F.; Łasińska, A.; Florjańczyk, Z.; Zygadło-Monikowska, E.; Affek, A. Crystallization and melting of PEO:LiTFSI polymer electrolytes investigated simultaneously by impedance spectroscopy and polarizing microscopy. *Electrochimica Acta* **2005**, *50*, 3969–3977, DOI: 10.1016/j.electacta.2005.02.053.

(3) Lunkenheimer, P.; Loidl, A. Relaxation Dynamics of Poly(ethylene oxide). *Macromolecules* **2025**, *58*, 3547–3553, DOI: 10.1021/acs.macromol.5c00265.

(4) Lascaud, S.; Perrier, M.; Vallee, A.; Besner, S.; Prud'homme, J.; Armand, M. Phase Diagrams and Conductivity Behavior of Poly(ethylene oxide)-Molten Salt Rubbery Electrolytes. *Macromolecules* **1994**, *27*, 7469–7477, DOI: 10.1021/ma00103a034.

(5) Molinari, N.; Mailoa, J. P.; Kozinsky, B. Effect of Salt Concentration on Ion Clustering and Transport in Polymer Solid Electrolytes: A Molecular Dynamics Study of PEO–LiTFSI. *Chemistry of Materials* **2018**, *30*, 6298–6306, DOI: 10.1021/acs.chemmater.8b01955.

(6) Tsamopoulos, A. J.; Wang, Z.-G. Ion Conductivity in Salt-Doped Polymers: Combined Effects of Temperature and Salt Concentration. *ACS Macro Letters* **2024**, *13*, 322–327, DOI: 10.1021/acsmacrolett.3c00757.

(7) Niedzwiedz, K.; Wischnewski, A.; Pyckhout-Hintzen, W.; Allgaier, J.; Richter, D.; Faraone, A. Chain Dynamics and Viscoelastic Properties of Poly(ethylene oxide). *Macromolecules* **2008**, *41*, 4866–4872, DOI: 10.1021/ma800446n.

(8) Perrier, M.; Besner, S.; Paquette, C.; Vallée, A.; Lascaud, S.; Prud'homme, J. Mixed-alkali effect and short-range interactions in amorphous poly(ethylene oxide) electrolytes. *Electrochimica Acta* **1995**, *40*, 2123–2129, DOI: 10.1016/0013-4686(95)00151-4.

(9) Mongcopa, K. I. S.; Tyagi, M.; Mailoa, J. P.; Samsonidze, G.; Kozinsky, B.; Mullin, S. A.; Gribble, D. A.; Watanabe, H.; Balsara, N. P. Relationship between Segmental Dynamics Measured by Quasi-Elastic Neutron Scattering and Conductivity in Polymer Electrolytes. *ACS Macro Letters* **2018**, *7*, 504–508, DOI: 10.1021/acsmacrolett.8b00159.

(10) Bergstra, J.; Bardenet, R.; Bengio, Y.; Kégl, B. Algorithms for Hyper-Parameter Optimization. Advances in Neural Information Processing Systems. 2011.

(11) Abril-Pla, O.; Andreani, V.; Carroll, C.; Dong, L.; Fonnesbeck, C. J.; Kochurov, M.; Kumar, R.; Lao, J.; Luhmann, C. C.; Martin, O. A.; Osthege, M.; Vieira, R.; Wiecki, T.;

Zinkov, R. PyMC: A Modern and Comprehensive Probabilistic Programming Framework in Python. *PeerJ Computer Science* **2023**, *9*, DOI: 10.7717/peerj-cs.1516.
